# WRKY27‐RAP2.7 Regulatory Module Promotes Cold Tolerance via Modulation of Lignin Biosynthesis and Redox Homeostasis by Regulating *Cinnamyl Alcohol Dehydrogenase 7* and *Glutathione S‐Transferase F6*

**DOI:** 10.1111/pbi.70439

**Published:** 2025-11-17

**Authors:** Jing Qu, Peng Xiao, Yilei Wang, Tian Fang, Haowei Chen, Chunlong Li, Ji‐Hong Liu

**Affiliations:** ^1^ National Key Laboratory for Germplasm Innovation & Utilization of Horticultural Crops, College of Horticulture and Forestry Sciences Huazhong Agricultural University Wuhan China; ^2^ Hubei Hongshan Laboratory Wuhan China

**Keywords:** *Citrus ichangensis*, cold stress, lignin biosynthesis, RAP2, ROS scavenging, WRKY

## Abstract

Cold stress adversely affects plant growth and development, significantly limiting fruit yield and quality in citrus. Ichang papeda (*Citrus ichangensis*), a cold‐tolerant citrus species, serves as a valuable genetic resource for studying cold adaptation, yet the key genes and their modes of action underlying the cold stress response remain largely unexplored. In this study, we identified CiWRKY27 as a critical positive regulator of cold tolerance in Ichang papeda. Through DNA affinity purification sequencing (DAP‐seq) and RNA‐sequencing analyses we uncovered 717 potential target genes of CiWRKY27, many of which are associated with stress adaptation. Further investigation revealed that CiWRKY27 directly activated *CiCAD7* and *CiGSTF6* by binding to W‐box elements in their promoters, thereby enhancing lignin biosynthesis and reactive oxygen species (ROS) detoxification to confer cold tolerance. Functional assays confirmed that both *CiCAD7* and *CiGSTF6* contributed positively to cold tolerance. Additionally, CiRAP2.7, an RAP2 (AP2/ERF) transcription factor, physically interacted with CiWRKY27 to synergistically amplify the activation of *CiCAD7*. Intriguingly, CiRAP2.7 was itself regulated by CiWRKY27 and functioned as a transcriptional activator of *CiCAD7* for rendering cold tolerance. Taken together, our findings demonstrate that CiWRKY27 works, alone or together with CiRAP2.7, to modulate cold tolerance by regulating *CiCAD7*‐mediated lignin biosynthesis and *CiGSTF6‐*dependent ROS scavenging. This study gains valuable insights into the molecular regulation of lignin biosynthesis and ROS homeostasis under cold stress, and offers a promising molecular module for improving cold tolerance in citrus and other crops.

## 1 Introduction

Citrus is one of the most economically important fruit crops, serving as a key nutritional source while contributing significantly to global agriculture. However, as a fruit tree of tropical origin, most commercial citrus cultivars are vulnerable to cold stress, which has been frequently reported to cause immense restrictions on plant growth and development, fruit yield, and quality of citrus (Dahro et al. 2023; Ding et al. 2024). Ichang papeda (*Citrus ichangensis*) displays remarkable cold tolerance, making it an ideal plant for deciphering cold adaptation mechanisms and identifying key cold‐responsive genes for molecular breeding (Yang et al. 2017; Wang et al. 2025). Despite its potential, there is a considerable knowledge gap in understanding the molecular regulatory mechanisms underlying the remarkable freezing tolerance of Ichang papeda.

In the presence of cold stress, the stimuli signal is perceived by receptors located on the plasma membrane of plant cells, followed by alterations in membrane fluidity and conformational changes in membrane proteins. Except for the cell membrane, the cell wall, as the external barrier and mechanical support of plant cells, effectively prevents ice formation within the membrane and is essential for the survival of plants during freezing (Ghosh and Xu 2014). Lignin, a phenylpropanoid‐based polymer, is a crucial component of the cell wall and plays an essential role in withstanding various external environmental stimuli to maintain plant cell integrity and viability (Han et al. 2022). Lignin is actively involved in response to cold stress as it influences the flexibility of cell walls to accommodate ice crystal growth and mitigate cellular damage due to dehydration during the cold acclimation process (Ji et al. 2015; dos Santos et al. 2015). The biosynthesis of lignin from phenylalanine is catalysed by an orchestrated cascade of enzymes, in which cinnamyl alcohol dehydrogenase (CAD) serves as the key enzyme that facilitates the final step of monolignol biosynthesis required for specialised lignin polymer biosynthesis (Li, Guo, et al. 2024). The functions of *CADs* in modulating lignin synthesis have been illustrated in several plants under stressful conditions. For instance, *EjCAD1* and *EjCAD3* of loquat were identified as the potential key genes associated with chilling‐induced lignification (Ge et al. 2017; Liu et al. 2019). A total of 50 *StCAD* genes were identified in potato, the majority of which were proven to be upregulated in response to cadmium stress (Yang et al. 2024). Very recently, *OsCAD6* of rice was shown to be induced by drought and may contribute to the enhanced drought tolerance in transgenic plants overexpressing *OsDof12* (Shim et al. 2025). In cassava, activation of *MeCAD15* by MeRAV5 resulted in an increased lignin content and enhanced drought tolerance (Yan et al. 2021). Nevertheless, the intricate regulatory mechanisms underlying the cold‐induced *CAD* expression and lignin synthesis remain largely unknown.

It has been well documented that cold stress triggers a sharp accumulation of reactive oxygen species (ROS), which has been proposed as one of the earliest cellular processes when plants are exposed to cold stress (Choudhury et al. 2016). Therefore, the orchestration of cell wall rigidity and redox homeostasis is critical to obtaining plant cold tolerance. Glutathione S‐transferases (GSTs) are a superfamily of multifunctional dimeric enzymes that play a critical role in scavenging ROS. There are 14 distinct classes of GSTs in plants, among which GSTUs and GSTFs are the most important types (Qi et al. 2018). The functional roles of GSTUs in response to low temperature stress have been widely confirmed in previous studies. For instance, *JrGSTTau1* of *Juglans regia* was identified as improving cold tolerance by promoting ROS scavenging (Yang et al. 2016). *PtrGSTU17* from trifoliate orange was reported to be involved in PtrERF9‐mediated cold tolerance via facilitating ROS scavenging (Zhang et al. 2021). In tea plants, the CsCIPK11‐CsGSTU23 module plays a central role in modulating antioxidant capacity and enhancing cold tolerance (Di et al. 2024). Nonetheless, the precise function and the upstream transcriptional regulators of most GSTFs in cold tolerance remain scarcely elucidated.

Transcriptional reprogramming of numerous stress‐responsive genes constitutes a highly conserved and sophisticated strategy for cold stress adaptation, in which transcription factors (TFs) are indispensable components to orchestrate the upstream signalling transduction and downstream stress response. Central to this process is the highly conserved regulatory module composed of INDUCER OF CBF EXPRESSION 1 (ICE1) and C‐repeat (CRT) binding factors (CBFs) identified across different plant species (Kidokoro et al. 2022). In this pathway, the ICE1 regulates and activates the *CBF* genes. As a result, the CBFs transcriptionally regulate numerous *cold‐regulated* (*COR*) genes through binding to the CRT/DRE elements in their promoters, initiating diverse downstream responses that are involved in the modulation of cold tolerance (Ding and Yang 2022). In addition to this pathway, several other TFs have also been proposed to make a critical contribution to the cold stress response and adaptation through transcriptional regulation. Among these TFs in plants, WRKY and ERF/AP2 TFs play a crucial role in plant responses to cold stress (Tang et al. 2022; Zhang et al. 2022; Xu et al. 2024; Yang, Fang, et al. 2025). Of note, some members of the WRKY and ERF/AP2 families have been shown to regulate the biosynthesis of lignin and the development of secondary cell walls. For example, WRKY46 in pear fruit directly activates *NAC187* expression, regulating *CCR* gene transcription and contributing to lignin accumulation (Cheng et al. 2025). In *Arabidopsis*, RAP2.6 plays a crucial role in the continuous production of lignin and the subsequent wound‐healing process (Xu et al. 2024). MdERF61 in apple roots repressed mdm‐miR397d expression, leading to increased MdLAC7b transcripts and enhanced lignin deposition during *F. solani* infection (Zhou et al. 2024). Although the TFs can work alone to regulate their target genes, accumulating evidence indicates that TFs from the same or different families interact to form protein complexes, which alter the DNA binding specificity and affinity of the interacting TFs and influence transcriptional activation activity efficiency (Bemer et al. 2017; Liu, Gao, et al. 2022). For instance, GhWRKY41 formed a homodimer that regulated the expression of *GhC4H* and *Gh4CL*, enhancing resistance to *Verticillium dahliae* in cotton (Xiao et al. 2023). StWRKY20 formed a MYB‐WRKY complex with StMYB168 to regulate lignin monomer synthesis during wound healing in potato tubers (Yang, Wang, et al. 2025). In tea plants, CsWRKY13 interacted with CsPAT1 to antagonistically regulate the expression of *CsPAL* and *CdC4H*, thereby modulating lignin accumulation (Li, Zhou, et al. 2024). Despite these progresses, the roles of specific TFs from the IIe WRKY and ERF/AP2 VII subgroups in lignin biosynthesis and the cold response have never been reported. Another raised question is whether WRKY and AP2/ERF proteins can form a complex to regulate lignin synthesis in response to cold stress.

In this study, we showed that CiWRKY27 acts as a positive regulator of Ichang papeda freezing tolerance. The DNA affinity purification sequencing approach, combined with RNA‐sequencing analysis, revealed that CiWRKY27 directly upregulates *CiCAD7*, which encodes cinnamyl alcohol dehydrogenase, a key enzyme involved in lignin biosynthesis, as well as *CiGSTF6*, which encodes a glutathione S‐transferase involved in reactive oxygen species scavenging. Furthermore, we demonstrated that CiWRKY27 physically interacts with and also regulates the ERF/AP2 family transcription factor CiRAP2.7. The CiWRKY27‐CiRAP2.7 complex coordinately regulates the expression of *CiCAD7*, thereby increasing lignin accumulation. Taken together, our findings advance the current understanding of the molecular regulation of lignin biosynthesis in plants under cold stress and provide an important molecular module that holds promise for improving plant adaptability and cold tolerance.

## 2 Results

### 2.1 CiWRKY27 Was a Nuclear Protein With Transcriptional Activation Activity

In our previous study, CiWRKY27 in the IIe group was substantially induced by cold (Qu et al. 2024). However, further exploration is necessary to elucidate the biological function and regulatory mechanism of CiWRKY27 in cold response. In this study, the transcript level of *CiWRKY27* under exogenous hormone treatments and cold exposure (4°C) was reassessed by RT‐qPCR. The mRNA abundance of *CiWRKY27* was significantly, once or at several time points, induced by ABA, BR, JA, SA (Figure S1). In addition, *CiWRKY27* transcript level exhibited a notable increase after 24 h of cold treatment, reaching its peak at 120 h (Figure S2a). *CiWRKY27* was ubiquitously expressed in various tissues, with the highest transcription levels in the pulp and stem (Figure S2b). Subcellular localisation analysis revealed a significant overlap between the nuclear marker mCherry and the CiWRKY27‐YFP signals, indicating that CiWRKY27 was a nuclear protein (Figure S2c).

To determine whether CiWRKY27 possesses the transcription activation activity, the full‐length CDS (CiWRKY27‐FL) and various truncated fragments (CiWRKY27‐ΔC/TF/ΔN) of CiWRKY27 were introduced into the *AH109* yeast strain (Figure S2d,e). The yeast cell only transformed with either CiWRKY27‐ΔC or CiWRKY27‐ΔN activated the reporter gene expression (conferring GUS activity), whereas those transformed with CiWRKY27‐FL or CiWRKY27‐TF did not. The results indicate that both the N‐terminal and C‐terminal regions of CiWRKY27 are essential for its transcriptional function. Moreover, the dual‐LUC reporter (DLR) assay demonstrated that tobacco expressing CiWRKY27 exhibited higher LUC activity compared to the negative control (Figure S2f,g). Taken together, these findings suggest that CiWRKY27 is a cold‐induced nucleus‐localised protein with transcriptional activation activity.

### 2.2 Overexpression of *CiWRKY27* Led to Enhanced Cold Tolerance in Transgenic Plants

**FIGURE 1:**
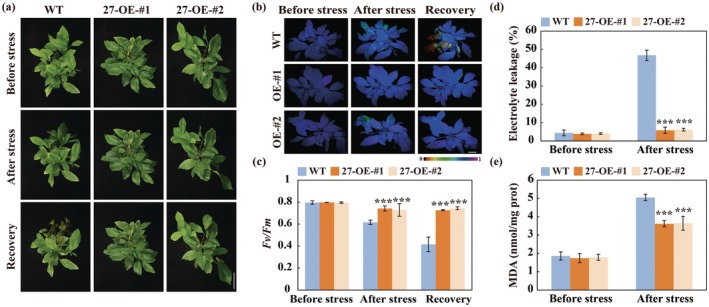
Overexpression of *CiWRKY27* confers enhanced cold tolerance in transgenic lemon. (a) Phenotype of lemon transgenic lines (27‐OE‐#1 and 27‐OE‐#2) and WT before and after cold treatment (4 h at −2°C, 8 h at −4°C), followed by recovery for 1 day at ambient environment. Scale bars = 5 cm. (b–e) Chlorophyll fluorescence imaging (b), *Fv/Fm* ratios (c), electrolyte leakage (EL) (d) and malondialdehyde (MDA) content (e) of tested lines before and after the cold treatment. Error bars indicate ±SD (*n* = 3). Asterisks indicate significant differences between the WT and transgenic plants under the same growth conditions (***p < 0.001).

We previously demonstrated that silencing of *CiWRKY27* by virus‐induced gene silencing (VIGS) markedly reduced cold resistance of Ichang papeda (Qu et al. 2025). To further explore the function of *CiWRKY27* in cold tolerance, we overexpressed *CiWRKY27* in lemon, one of the most cold‐sensitive citrus species (Figure S3). Under normal conditions, both the wild type (WT) and transgenic lines (27‐OE‐#1 and 27‐OE‐#2) exhibited normal and healthy growth. However, when subjected to freezing temperatures at −2°C for 4 h and −4°C for 8 h, the WT plants wilted and perished, while the transgenic plants remained alive (Figure 1a). Consistent with the phenotypic observations, the transgenic plants displayed better chlorophyll fluorescence and higher *Fv/Fm* ratios (Figure 1b,c), along with reduced electrolyte leakage (EL) and malondialdehyde (MDA) levels (Figure 1d,e), compared to the WT following the cold treatment.

To further examine the function of *CiWRKY27*, we generated transgenic tobacco lines overexpressing *CiWRKY27* (#2 and #4) and assessed their cold tolerance in comparison with the wild‐type plants (Figure S4). Under normal growth conditions, there were no morphological differences between the WT and transgenic plants. However, following freezing treatment (4 h at 4°C, 8 h at −2°C), the transgenic plants displayed a more pronounced cold‐tolerance phenotype compared to the WT (Figure S5a). In addition, stronger chlorophyll fluorescence, higher *Fv/Fm* ratios and survival rates (Figure S5b–d), but significantly lower levels of MDA and EL (Figure S5e,f) and markedly less accumulation of H_2_O_2_ and $O_{2}^{.-}$ were detected in the transgenic plants than in the WT under cold treatment (Figure S5g). Collectively, these findings indicate that the overexpression of *CiWRKY27* significantly elevated cold tolerance of the transgenic plants.

### 2.3 Identification of CiWRKY27 Target Genes by Transcriptome and DAP‐Seq Analyses

**FIGURE 2:**
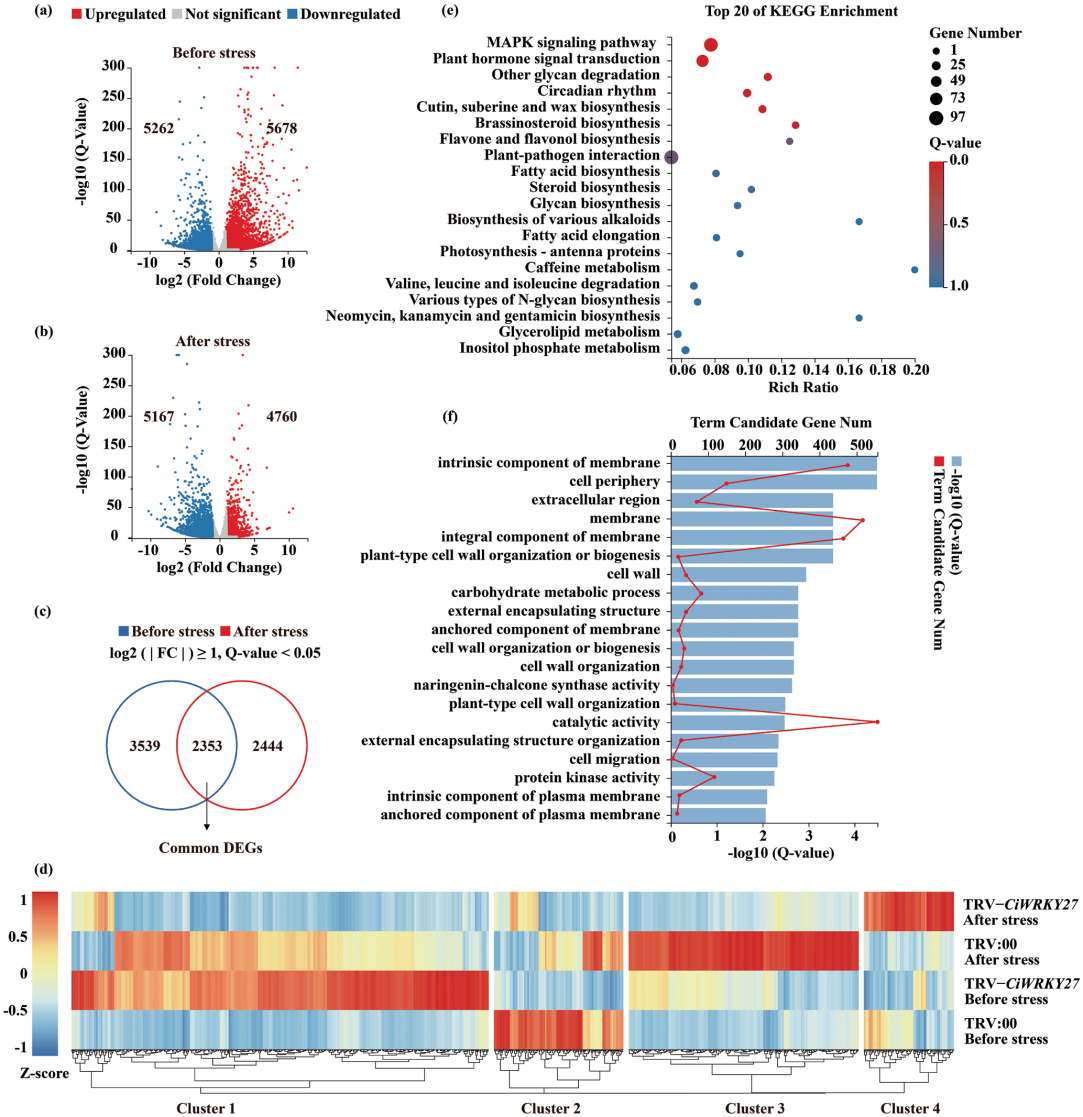
Transcriptome profiles of CiWRKY27‐regulated genes. (a‐b) Volcano plots of the differentially expressed genes (DEGs) in TRV‐*CiWRKY27* compared to TRV:00 control before (a) and after (b) the cold treatment. The DEGs from the RNA‐seq were determined based on the criteria: Fold change ≥ 2.0, *Q*‐value < 0.05. Up‐regulated and down‐regulated genes are represented by red and blue circles, respectively, while the grey ones indicate those without significant change. (c) Venn diagrams showing the DEGs regulated by both cold and CiWRKY27. Overlapping represents the DEGs in leaves of TRV‐*CiWRKY27* plants compared to TRV control before (blue) and after (red) cold treatment. (d) Clustering analysis of 1319 DEGs whose promoters contained the W‐box elements, based on the Log_2_ (TPM) values of the DEGs. (e, f) The top 20 enriched KEGG pathways (e) and GO terms (f) in the DEGs regulated by CiWRKY27.

To gain a deeper understanding of the molecular mechanisms underlying cold stress response and to identify the target genes regulated by *CiWRKY27*, comparative transcriptome analysis was conducted through deep sequencing (RNA‐seq) using leaves from *CiWRKY27*‐VIGS plants (TRV‐*CiWRKY27*) and TRV:00 control before and after cold treatment (8 h at −4°C, Qu et al. 2025). We obtained a total of 43.04 million reads after trimming, with 98.2% of which were mapped to unique loci in the reference genome (Table S3). Based on the threshold fold change ≥ 2 and *Q*‐value < 0.05, 10 940 differentially expressed genes (DEGs) were identified before the cold treatment, of which 5262 were downregulated and 5678 were upregulated in the TRV‐*CiWRKY27* line relative to the TRV:00 control (Figure 2a). Upon exposure to the cold treatment, 5167 downregulated and 4760 upregulated genes were differentially expressed in the TRV‐*CiWRKY27* line (Figure 2b). A total of 2353 DEGs that exhibited prominent variations between normal conditions and low temperature were identified in the VIGS line. Of them, 1319 genes were found to contain W‐box elements in their promoters, suggesting that they might be possibly regulated by CiWRKY27 (Figure 2c). We subsequently performed the cluster analysis and grouped the 1319 DEGs into four clusters (Clusters 1–4) and focused on candidate targets which were induced by cold (Figure 2d). The KEGG analysis revealed marked enrichment of several processes, such as the ‘MAPK signaling pathway’, ‘plant hormone signal transduction’, ‘circadian rhythm’ and ‘cutin, suberine and wax biosynthesis’. GO analysis indicated that the DEGs were involved in several metabolic pathways related to membrane and cell wall organisation, such as ‘intrinsic component of membrane’ and ‘plant‐type cell wall organization or biogenesis’, highlighting the essential protective roles of cell walls and membranes in cold tolerance (Figure 2e,f).

**FIGURE 3:**
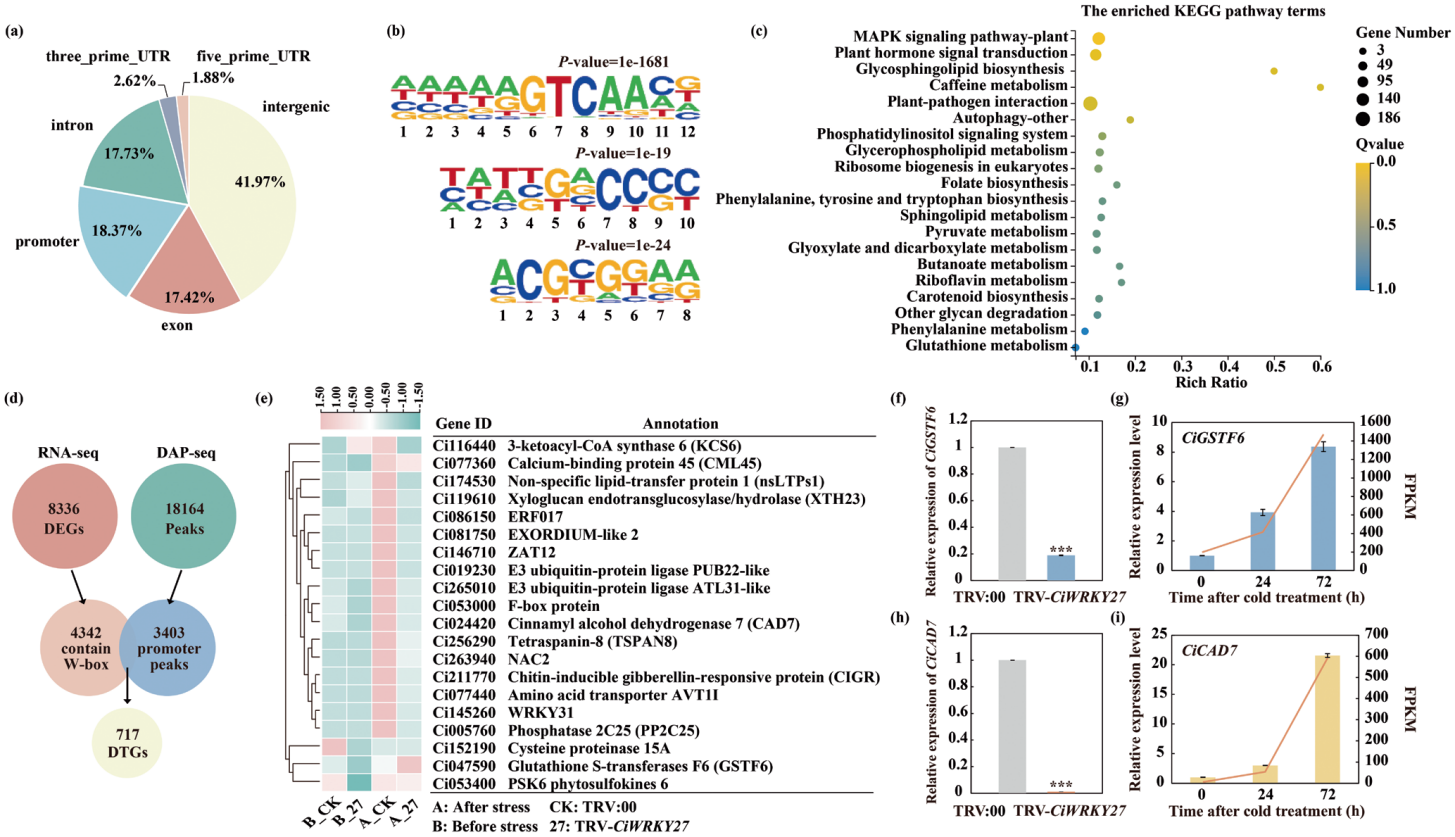
Genome‐wide identification of CiWRKY27 binding sites, as revealed by DAP‐seq. (a) Distribution of CiWRKY27 binding peaks across the genomic features. (b) DNA logos of enriched DNA binding sites for CiWRKY27, as determined by MEME (version 5.5.2) online tools. (c) The top 20 enriched KEGG pathways of CiWRKY27‐bound genes identified through DAP‐seq. (d) Venn diagram showing the overlap between genes bound by CiWRKY27 in DAP‐seq and differentially expressed genes (DEGs) regulated by CiWRKY27 from RNA‐seq. (e) Expression of top 20 CiWRKY27‐binding candidate genes in TRV:00 and TRV‐*CiWRKY27* plants before and after cold treatment. (f–i) The expression level of *CiGSTF6* (f, g) and *CiCAD7* (h, i) in TRV:00 and TRV‐*CiWRKY27* plants before cold treatments, as well as in wild‐type plants exposed to cold treatment at different time points (0, 24, 72 h), assessed by RT‐qPCR (columns) and RNA‐seq (lines). Error bars indicate ±SD (*n* = 3). Asterisks indicate significant differences between the WT and transgenic plants under the same growth conditions (***p < 0.001).

To enhance our understanding of CiWRKY27‐mediated transcriptional regulation of cold tolerance, DAP‐seq was employed to unravel binding sites of CiWRKY27 at a genome‐wide scale. As a result, 50.18 million reads were obtained, among which 99.5% were mapped to unique loci in the reference genome of Ichang papeda. A total of 18 164 enriched peaks, corresponding to 11 352 genes, were consistently detected and deemed as the high‐confidence binding regions of CiWRKY27 (Table S4). The binding sites ranged from 165 to 2048 bp in size (Figure S6a), and were predominantly centred on transcriptional start sites (TSS) (Figure S6b). Moreover, 18.37% of the binding sites were located within the promoter regions (up to 2‐kb upstream from the TSS). In addition, it was observed that other binding sites were located in the 5′‐UTRs (1.88%), 3′‐UTRs (2.62%), exons (17.42%), introns (17.73%) and intergenic regions (41.97%) (Figure 3a). To gain further insight into the DNA‐binding properties of CiWRKY27, a de novo motif prediction was performed by MEME tools (version 5.5.2) based on the CiWRKY27‐binding regions identified in DAP‐seq. Consequently, three enriched motifs were identified, including N (A/C/T) (A/T) A (A/G) GTCAACN, (C/T) (A/T) T (C/G/T) G (A/G) CCCC and ACG (C/T) GGA (A/G), which were detected in 2933, 505 and 506 CiWRKY27‐binding peaks, respectively (Figure 3b). Notably, the first enriched motif harboured the core sequence TTGA (C/T), a canonical motif recognised by WRKYs (Rushton et al. 2010; Javed and Gao 2023). KEGG analysis of the genes corresponding to the 3404 peaks demonstrates that the top pathways enriched were those related to ‘MAPK signaling pathway’, ‘plant hormone signal transduction’, ‘glycosphingolipid biosynthesis’, ‘caffeine metabolism’ and ‘plant‐pathogen interaction’ (Figure 3c). We then performed an integrative analysis of DAP‐seq and RNA‐seq results, and found that a total of 717 genes were overlapped between them, suggesting that they are possibly direct target genes of CiWRKY27 (Figure 3d). Furthermore, the top 20 candidate genes that exhibited the largest changes in FPKM values after cold treatment in Ichang papeda relative to the TRV:00 control plants were selected for further analysis (Figure 3e). To elucidate the potential regulatory pathways responsible for the function of *CiWRKY27* in cold tolerance, we particularly focused on identifying the functional genes that might play a direct role in protecting plant cells and counteracting cold stress. Two of these genes, *glutathione S‐transferases F6* (*CiGSTF6*) and *cinnamyl alcohol dehydrogenase 7* (*CiCAD7*), were substantially induced by cold stress, but drastically downregulated in the TRV‐*CiWRKY27* plants in comparison with the TRV:00 control (Figure 3f–i). Therefore, they were selected for further investigation.

### 2.4 CiWRKY27 Activated *CiGSTF6* and *CiCAD7* by Directly Binding to the Promoters

**FIGURE 4:**
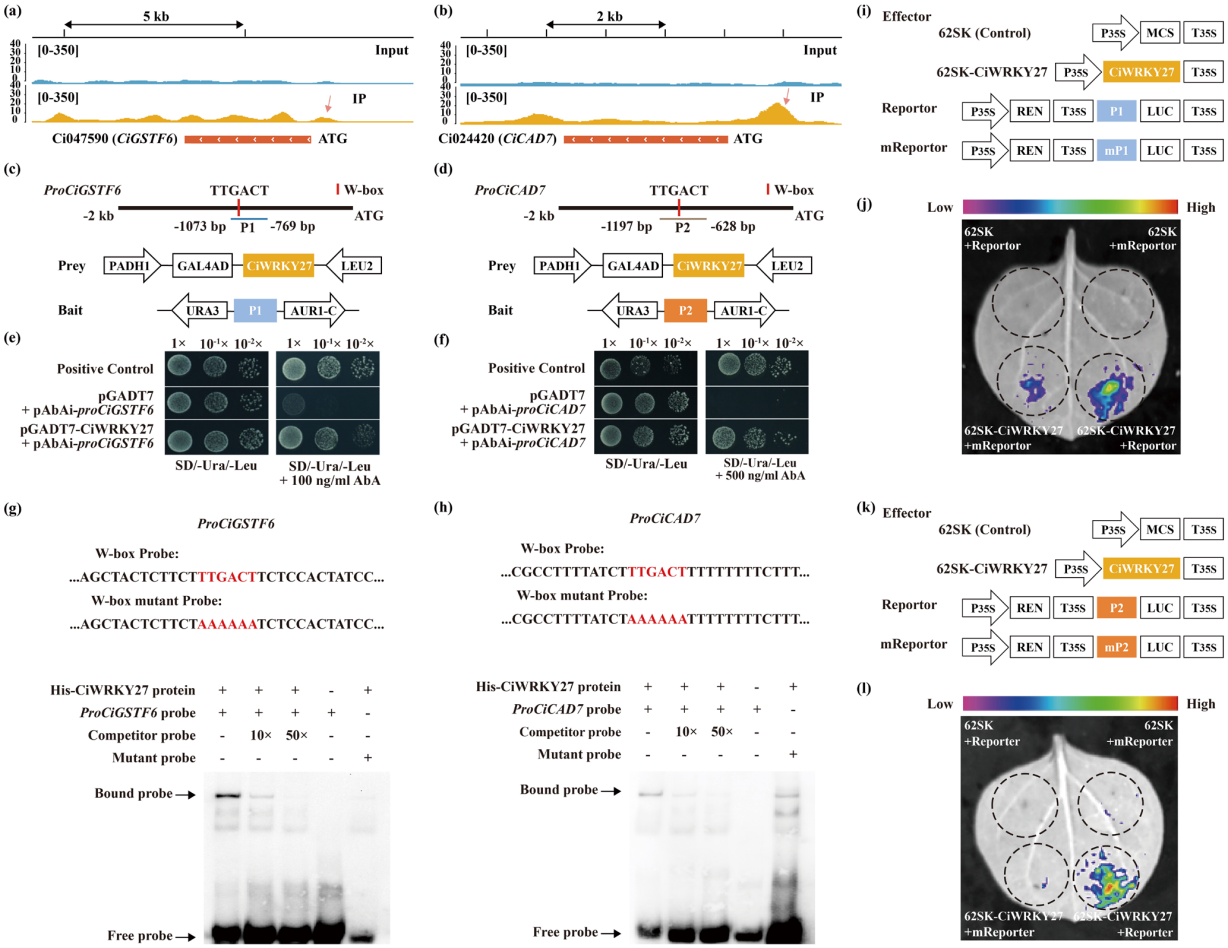
CiWRKY27 directly bound to and activated the promoters of *CiGSTF6* and *CiCAD7*. (a, b) CiWRKY27 binding peaks (IP) and negative control (Input) over the *CiGSTF6* (a) and *CiCAD7* (b) loci as determined by DAP‐seq. [0–350] represents the scale of binding intensity as reflected by the heights of the peak. (c, d) Schematic diagrams of *CiGSTF6* (c) and *CiCAD7* (d) promoters, in which the red rectangles indicate the W‐box elements. pGADT7‐CiWRKY27 served as the prey and pAbAi‐*proCiGSTF6* or pAbAi‐*proCiCAD7* were used as the baits, respectively. (e, f) Growth of yeast cells co‐transformed with positive control (p53‐AbAi + pGAD‐p53), baits + prey or negative control (bait + pGADT7) on SD/‐Ura/−Leu medium added with or without AbA. (g, h) EMSA assay shows that CiWRKY27 directly bound to the W‐box elements in the *CiGSTF6* (g) and *CiCAD7* (h) promoters. Recombinant purified HIS‐CiWRKY27 protein was incubated with biotin‐labelled probes containing the wild‐type or mutated W‐box elements. Unlabeled probes were used as a DNA competitor. (i–l) Schematic diagrams (i, k) of the effector (62SK‐CiWRKY27) and reporter constructs (0800‐*proCiGSTF6* and 0800‐*proCiCAD7* or their counterparts with mutated W‐box elements) for dual‐LUC transient expression assay and representative bioluminescence imaging of leaves infiltrated with the effector and 0800‐*proCiGSTF6* (j) or 0800‐*proCiCAD7* (l). MCS, multiple cloning sites. P_35S_, CaMV *35S* promoter. T_35S_, CaMV *35S* terminator. LUC, firefly luciferase. REN, *Renilla* luciferase. Error bars indicate ±SD (*n* = 3).

Both *CiGSTF6* and *CiCAD7* exhibited enriched binding sites in their promoter regions in the DAP‐seq profile (Figure 4a,b). To verify whether they were the downstream targets of CiWRKY27, their promoter fragments were integrated into the pAbAi vectors, while the CDS of CiWRKY27 was fused with the activation domain (AD) of GAL4 (Figure 4c,d). Y1H assay showed that only the yeast ( *Saccharomyces cerevisiae* ) cells transformed with the vectors pGADT7‐CiWRKY27 and each of pAbAi‐pro*CiGSTF6* and pAbAi‐pro*CiCAD7* survived on the selective medium supplemented with aureobasidin A (Figure 4e,f), indicating that CiWRKY27 interacts with the promoters of *CiGSTF6* and *CiCAD7* in yeast. Furthermore, EMSA was conducted to verify the interaction. Incubation of purified HIS‐CiWRKY27 protein and biotin‐labelled probes derived from the promoter fragments of *CiGSTF6* and *CiCAD7* (Figure 4g,h) gave rise to a band shift, which was prominently impaired by adding unlabeled DNA competitors in a dose‐dependent manner. However, mutation of the W‐box motif in the promoter fragment of *CiGSTF6* and *CiCAD7* completely abolished the band shift. These results demonstrate that CiWRKY27 specifically binds to the promoters of *CiGSTF6* and *CiCAD7* in vitro. DLR assay was then used to investigate the transcriptional activation or repression effects of CiWRKY27. The results showed that co‐expression of CiWRKY27 and the promoters of *CiGSTF6* (Figure 4i,j) and *CiCAD7* (Figure 4k,l) fused to the LUC reporter gene led to significant activation of the LUC activity in *Nicotiana benthamiana* cells, while the activation was not observed when the W‐box motifs in the promoter sequences were mutated, in comparison with the control. Collectively, these findings indicate that CiWRKY27 directly binds to the promoters of *CiGSTF6* and *CiCAD7* and activated their expression.

### 2.5 CiWRKY27 Regulated Lignin Biosynthesis and ROS Scavenging

**FIGURE 5:**
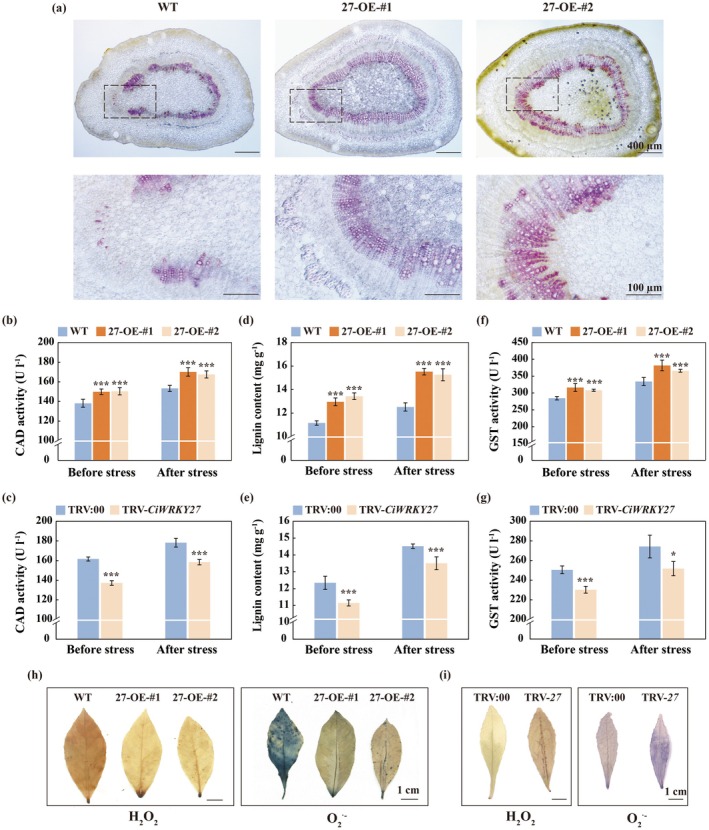
CiWRKY27 regulated lignin biosynthesis and ROS scavenging for promoting cold tolerance. (a) The histochemical staining of lignin deposition in the stems from wild type (WT) and *CiWRKY27*‐overexpressing lines under normal growth conditions. The bottom panels are zoomed‐in images marked as rectangles in the corresponding images at upper panels. (b, c) CAD activities (b, c), lignin content (d, e) and GST activities (f, g) in the transgenic lemon plants (b, d, f) and VIGS plants (TRV‐*CiWRKY27*, c, e, g), along with the WT and TRV:00 control, before and after cold stress. (h, i) Histochemical staining with 3, 3′‐diaminobenzidine (left panel) and nitro blue tetrazolium (right panel) of WT, OE‐*CiWRKY27* lemons (h) or TRV control (TRV:00), Ichang papeda VIGS (TRV‐*CiWRKY27*) plants (i) after cold treatment. Error bars indicate ±SD (*n* = 3). Asterisks indicate significant differences between groups (*p < 0.05; ***p < 0.001).

Cinnamyl alcohol dehydrogenase (CAD) is a pivotal enzyme that catalyses the final step of monolignol biosynthesis. As *CiCAD7* is a target gene of CiWRKY27, efforts were made to investigate whether CiWRKY27 regulated *CiCAD7*‐mediated lignin biosynthesis for cold tolerance. Transcript levels of *CiCAD7* were elevated in the transgenic plants overexpressing *CiWRKY27*, but were significantly reduced in the VIGS line, when compared to the WT and TRV:00, respectively (Figure S3 and Figure 3h). Phloroglucinol‐HCl staining revealed a significantly higher accumulation of lignin in the stems of *CiWRKY27*‐overexpressing lines when compared to the WT (Figure 5a). Moreover, the activity of CAD (Figure 5b,c) and lignin content (Figure 5d,e) were markedly elevated in the overexpressing lines, but prominently decreased in the VIGS lines relative to the WT and TRV:00. Meanwhile, cold exposure resulted in a dramatic increase in both the enzyme activity and lignin content in the transgenic overexpressing lines. Besides, in the wild type of Ichang papeda, cold exposure continuously increased CAD activity, which peaked at 120 h (Figure S7a). Similarly, lignin content showed a parallel trend: it increased slightly after 6 h, rose sharply and steadily from 24 h onward, and also reached its maximum at 120 h (Figure S7b).

On the other hand, the expression levels of *CiGSTF6* and GST activity were also detected in the transgenic plants. The transgenic lemon plants overexpressing *CiWRKY27* exhibited higher levels of *CiGSTF6* transcript and GST activity (Figure S3 and Figure 5f,g), which were otherwise substantially decreased in the VIGS line, relative to the WT or TRV:00 irrespective of cold treatment application. Since GST is responsible for ROS scavenging, we examined in situ accumulation of H_2_O_2_ and $O_{2}^{.-}$ in the tested plants (Figure 5h,i). Histochemical staining with DAB and NBT showed that both H_2_O_2_ and $O_{2}^{.-}$ were drastically decreased in the overexpressing lines, but evidently elevated in the VIGS lines, compared to the WT and TRV:00 control, largely consistent with the changes in the alteration of *CiGSTF6* transcript levels and GST activity. These results indicate that CiWRKY27 functioned to promote cold tolerance by regulating *CiCAD7*‐mediated lignin biosynthesis and *CiGSTF6*‐mediated ROS scavenging.

### 2.6 *CiCAD7* and *CiGSTF6* Functions Positively in Modulation of Cold Tolerance

**FIGURE 6:**
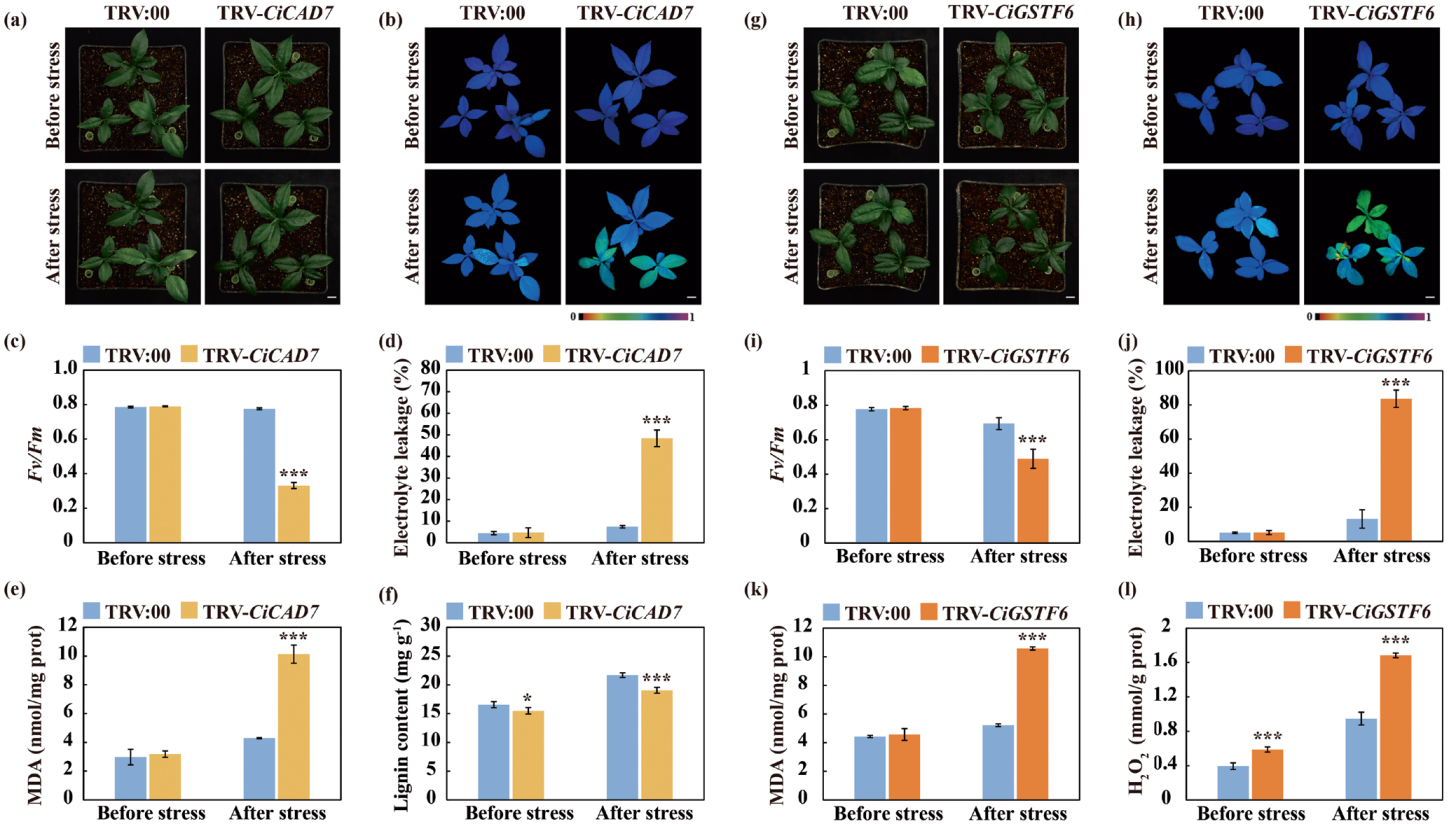
*CiCAD7* and *CiGSTF6* functioned positively in the modulation of cold tolerance. (a) Phenotype of TRV:00 control and VIGS plants (TRV‐*CiCAD7*) before and after cold treatment (8 h at −4°C). Scale bars = 1 cm. (b–e) Chlorophyll fluorescence imaging (b), *Fv*/*Fm* ratios (c), electrolyte leakage (d), MDA content (e) and lignin content (f) of tested plants before and after cold treatment. (g) Phenotype of TRV:00 control and VIGS plants (TRV‐*CiGSTF6*) before and after cold treatment (8 h at −4°C). Scale bars = 1 cm. (h–l) Chlorophyll fluorescence imaging (h), *Fv*/*Fm* ratios (i), electrolyte leakage (j), MDA content (k) and H_2_O_2_ content (l) of tested plants before and after cold treatment. Error bars indicate ±SD (*n* = 3). Asterisks indicate significant differences between TRV:00 and VIGS plants (TRV‐*CiCAD7* or TRV‐*CiGSTF6*) under the same growth condition (*p < 0.05; ***p < 0.001).

As *CiCAD7* and *CiGSTF6* were direct targets of WRKY27, subsequent experiments were conducted to characterise their role in cold tolerance. To answer this question, the VIGS was employed to silence *CiCAD7* and *CiGSTF6* to investigate their function in cold tolerance (Figures S8 and S9). The cold tolerance assessment revealed that the VIGS plants with knockdown of either *CiCAD7* or *CiGSTF6* exhibited more serious plant damage relative to the TRV:00 control (Figure 6a,g). In line with the plant phenotypes, weakened chlorophyll fluorescence and lower *Fv/Fm* ratios, but significantly elevated EL and MDA levels, were observed in the TRV‐*CiCAD7* and TRV‐*CiGSTF6* VIGS lines relative to their TRV:00 control (Figure 6b–e,h–k). In addition, the lignin levels were significantly decreased in the TRV‐*CiCAD7* plants, whereas H_2_O_2_ accumulation was substantially elevated in the TRV‐*CiGSTF6* lines compared to the TRV:00 control regardless of cold treatment (Figure 6f,l). These results demonstrate that both *CiCAD7* and *CiGSTF6* play positive roles in cold tolerance, which may be ascribed to promoting lignin biosynthesis and ROS scavenging, respectively.

### 2.7 CiRAP2.7 Interacted With and Was Regulated by CiWRKY27

**FIGURE 7:**
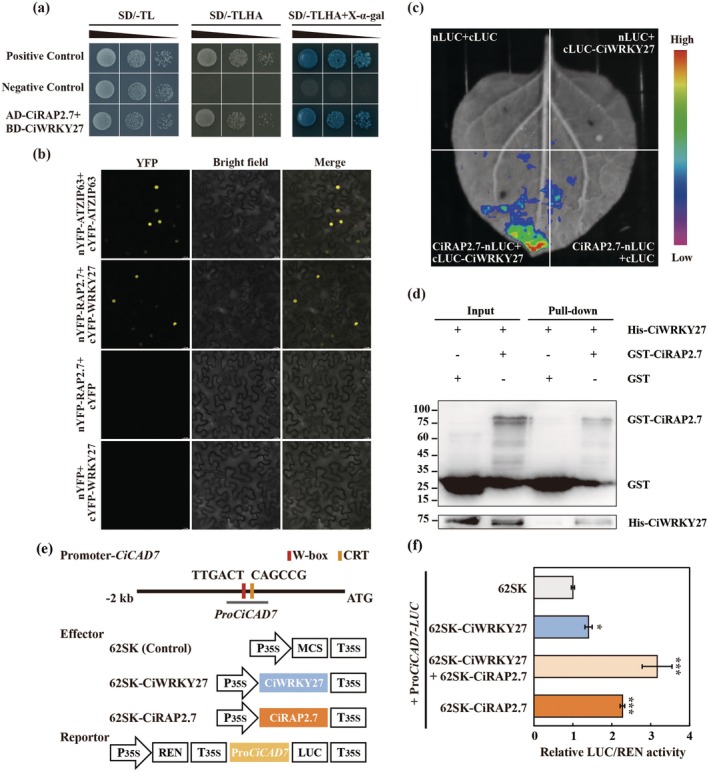
Interactions with CiRAP2.7 boosted transcriptional activation activity of CiWRKY27. (a) Growth of yeast cells co‐transformed with positive control (pGADT7‐T + pGBKT7‐53), negative control (pGADT7‐T + pGBKT7‐Lam) and AD‐CiRAP2.7 + BD‐CiWRKY27 on SD/−Trp/−Leu medium and SD/−Trp/−Leu/‐His/−Ade with or without X‐α‐gal (5‐Bromo‐4‐chloro‐3‐indolyl‐α‐D‐galactoside). (b) BiFC assay showing the interaction between CiWRKY27 and CiRAP2.7 in the nucleus. CiWRKY27 and CiRAP2.7 were fused to the C terminus or N terminus of yellow fluorescence protein (YFP). The YFP signals were observed under confocal microscopic. (c) LCI assay for verifying the interaction between CiWRKY27 and CiRAP2.7. CiWRKY27 and CiRAP2.7 were separately fused to the C terminus or N terminus of luciferease (LUC), and the resulting constructs (cLUC‐CiWRKY27, CiRAP2.7‐nLUC) were co‐infiltrated in *N*. *benthamiana* leaves. LUC illuminance was observed under the vivo plant imaging system. (d) Determination of interaction between CiWRKY27 and CiRAP2.7 by pull‐down assay. Recombinant GST‐CiRAP2.7 bound to the glutathione (GST) Sepharose beads was incubated with HIS‐CiWRKY27. The eluted proteins were detected by immunoblotting using anti‐GST and anti‐HIS antibodies, respectively. The protein incubated with the GST and HIS‐tag empty vector was served as a negative control. (e) Schematic diagrams of *CiCAD7* promoter and the schematic diagrams of effector and reporter constructs used for dual luciferase (LUC) transient expression assay. (f) LUC/REN ratios of *N*. *benthamiana* leaf infiltrated with CiWRKY27 and CiRAP2.7, alone or together, and the reporter vector made using *CiCAD7* promoter. LUC, firefly luciferase; REN, *Renilla* luciferase. Error bars indicate ±SD (*n* = 3). Asterisks indicate significant differences between groups (*p < 0.05; ***p < 0.001).

To illustrate the molecular mechanism of CiWRKY27 in conferring cold tolerance, Y2H screening was conducted to identify CiWRKY27 interacting proteins. A total of 82 randomly selected clones were sequenced (Figures S10 and S11), among which *CiRAP2.7*, encoding an AP2/ERF‐family transcription factor, was found to emerge as the predominant interactor of CiWRKY27. Point‐by‐point Y2H counter assays confirmed the interaction between CiRAP2.7 and CiWRKY27, as yeast cells co‐transformed with pGADT7‐CiRAP2.7 and pGBKT7‐CiWRKY27 displayed robust growth on QDO and activated X‐α‐gal (Figure 7a). BiFC assays showed that co‐infiltration of nYFP‐CiRAP2.7 and cYFP‐CiWRKY27 in *N. benthamiana* leaves led to recovery of YFP signals (Figure 7b). LCI assays showed that LUC activity was strongly reconstituted in the *N. benthamiana* leaves co‐expressing CiRAP2.7‐nLUC and cLUC‐CiWRKY27 (Figure 7c), suggesting that CiRAP2.7 associated with CiWRKY27 in planta. In addition, pull‐down assays indicated that GST‐CiRAP2.7 could pull down His‐CiWRKY27, but not GST alone, implying the direct interaction between CiRAP2.7 and CiWRKY27 in vitro (Figure 7d). In summary, these data strongly indicate that CiWRKY27 could interact with CiRAP2.7. Next, we investigated the effects of protein interaction on CiWRKY27‐mediated activation of downstream target genes by performing transient activation assays. Co‐expression of 62SK‐CiWRKY27 and 62SK‐CiRAP2.7 led to a greater activation of the LUC reporter gene driven by the promoter of *CiCAD7* compared to the expression of 62SK‐CiWRKY27 alone (Figure 7e,f), implying that interaction with CiRAP2.7 enhanced the activation activity of CiWRKY27.

**FIGURE 8:**
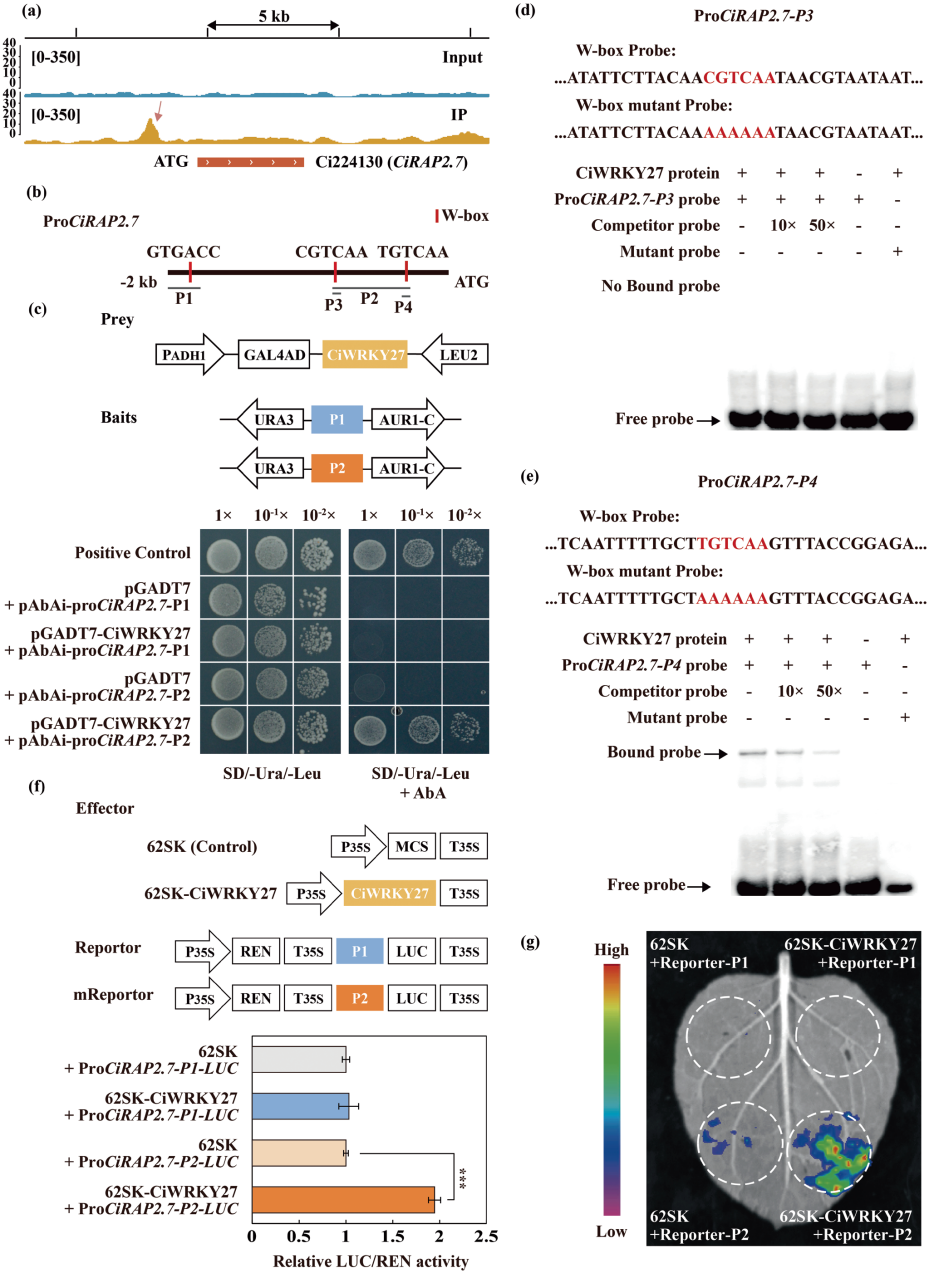
CiWRKY27 directly bound to and activated the promoter of *CiRAP2.7*. (a) CiWRKY27 binding peaks (IP) and negative control (Input) over the *CiRAP2.7* loci as determined by DAP‐seq. [0–350] represents the intensity of binding as reflected by the heights of the peak. (b) Schematic diagrams of *CiRAP2.7* promoter, in which the red rectangles indicate W‐box elements contained in the promoter. (c) Growth of yeast cells co‐transformed with positive control (p53‐AbAi + pGAD‐p53), baits + prey or negative control (bait + pGADT7) on SD/‐Ura/−Leu medium added without or with AbA. (d, e) EMSA assay shows that CiWRKY27 directly bound to the W‐box element in the P4 fragment of *CiRAP2.7* promoter. Recombinant HIS‐CiWRKY27 protein was purified and incubated with biotin‐labelled probes containing the wild‐type or mutated W‐box element. (f) Schematic diagrams represent the effector and reporter constructs used for dual luciferase (LUC) transient expression assay. MCS, multiple cloning sites. P_35S_, the promoter of CaMV *35S*. T_35S_, The terminator of CaMV *35S*. LUC, firefly luciferase. REN, *Renilla* luciferase. LUC/REN ratios of *N*. *benthamiana* leaf infiltrated with CiWRKY27 effector or SK empty vector control (EV) and the reporter vectors constructed with two fragments (P1 and P2) of *CiRAP2.7 promoter*. (g) Representative bioluminescence image of *N*. *benthamiana* leaf revealing the interaction between of CiWRKY27 and the *CiRAP2.7* promoter. Error bars indicate ±SD (*n* = 3). Asterisks indicate significant differences between groups (ns, no significant; *p < 0.05; ***p < 0.001).

As CiWRKY27 and CiRAP2.7 are two TFs we wanted to know whether they could regulate each other at transcriptional levels. To answer this question, we first analysed their promoter sequences and noticed that three potential W‐box sequences were present in the promoter of *CiRAP2.7*, whereas no AP2/ERF‐recognising motifs, including CRT or GCC‐box elements, were observed in the promoter of *CiWRKY27* (Figure S12). Interestingly, we identified a binding peak of CiWRKY27 in the promoter of *CiRAP2.7* based on the DAP‐seq data (Figure 8a), implying that CiWRKY27 may possibly regulate *CiRAP2.7*. We then conducted Y1H assays and found CiWRKY27 interacted with the *CiRAP2.7* promoter (Figure 8b,c). Moreover, EMSA further indicates that CiWRKY27 specifically bound to the W‐box motif closest to the start codon (Figure 8d,e). Dual LUC assays demonstrate that CiWRKY27 activated the reporter gene driven by the *CiRAP2.7* promoter fragment containing the recognised W‐box element (Figure 8f,g). Furthermore, we observed the transcript levels of *CiRAP2.7* were significantly upregulated in transgenic lemon plants overexpressing *CiWRKY27*, but exhibited marked downregulation in the TRV‐*CiWRKY27* plants (Figures S3 and S13). Collectively, these results indicate that *CiRAP2.7* functioned as a downstream target gene of CiWRKY27.

### 2.8 CiRAP2.7 Positively Regulates Cold Tolerance

To gain further insights into the function of CiRAP2.7, we conducted RT‐qPCR to assess its expression in response to cold treatment. As shown in Figure S14a, *CiRAP2.7* was found to be upregulated after 72 h treatment at 4°C. In addition, *CiRAP2.7* was most abundantly expressed in the stem, followed by the leaf and root, and the lowest expression levels were observed in the peel and pulp (Figure S14b). CiRAP2.7 was confirmed to be solely localised in the nucleus (Figure S14c). In addition, CiRAP2.7 exhibited the transcription activation ability, and the C‐terminal is crucial for the activation (Figure S14d,e). Meanwhile, the LUC activity was substantially and significantly elevated when 62SK‐CiRAP2.7 was infiltrated with 0800‐TATA reporter in comparison with the negative control (Figure S14f,g). Collectively, these results demonstrate that CiRAP2.7 is a cold‐responsive and nucleus‐localised transcriptional activator.

**FIGURE 9:**
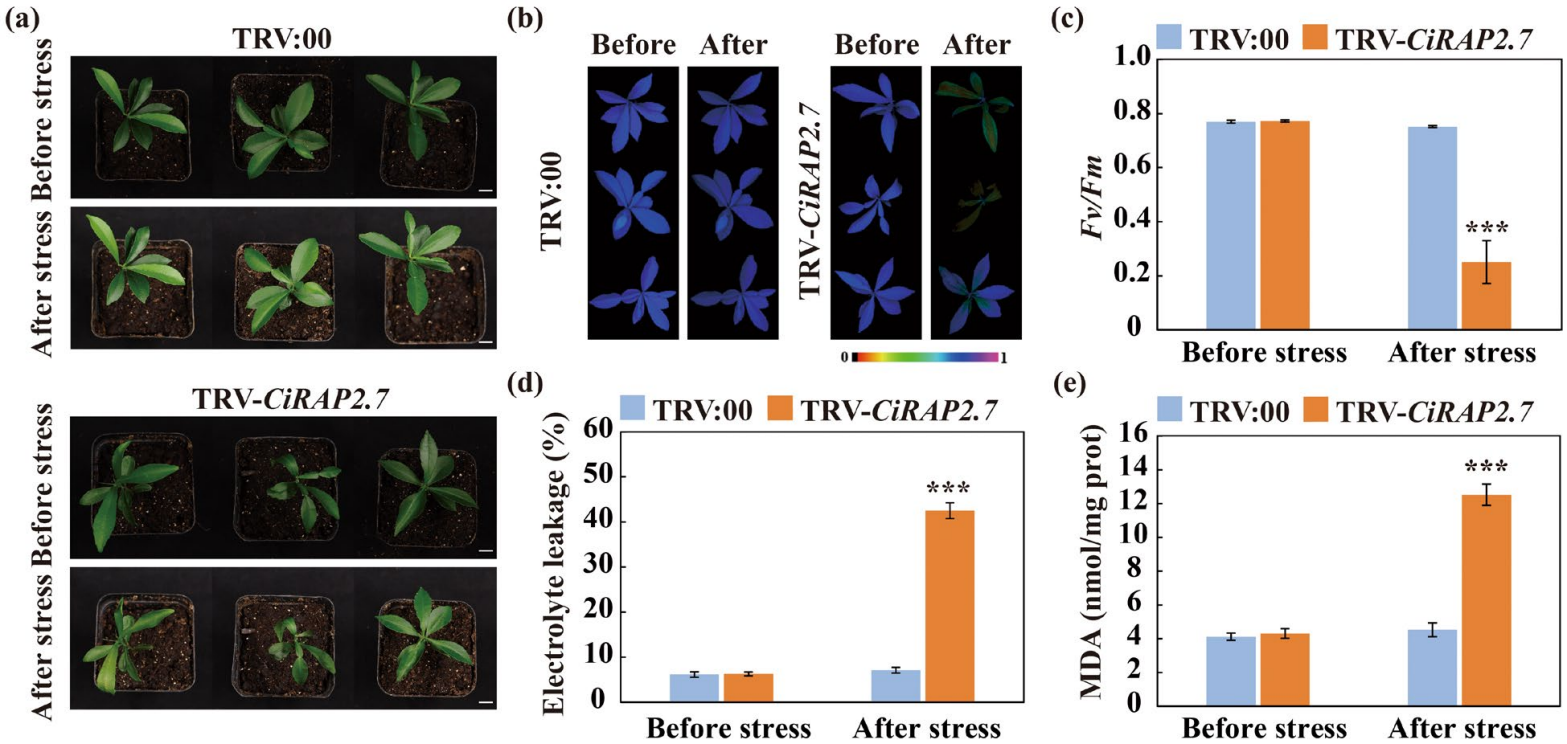
CiRAP2.7 positively regulated cold tolerance. (a) Phenotype of TRV:00 control and VIGS plants (TRV‐*CiRAP2.7*), measured before and after cold treatment (8 h at −4°C). Scale bars = 1 cm. (b–e) Chlorophyll fluorescence imaging (b), *Fv*/*Fm* ratios (c), electrolyte leakage (d) and MDA content (e) of TRV:00 control and VIGS plants before and after the cold treatment. The false colour scale between 0 and 1 is shown under the imaging. Error bars indicate ±SD (*n* = 3). Asterisks indicate significant differences between TRV:00 control and VIGS plants under the same growth condition (***p < 0.001).

Since CiRAP2.7 was induced by cold stress, we hypothesised that it may play a role in mediating cold tolerance. To test this hypothesis, *CiRAP2.7* in Ichang papeda was silenced by using VIGS (Figure S15). Compared to TRV:00 control, the TRV‐*CiRAP2.7* plants exhibited increased sensitivity to cold treatment, as evidenced by more serious leaf curling and waterlogging (Figure 9a). Furthermore, the VIGS plants exhibited lower chlorophyll fluorescence intensity and *Fv/Fm* ratios, together with significantly increased EL and MDA content, compared to the TRV control in the presence of cold stress (Figure 9b–e). These results indicated that silencing *CiRAP2.7* increases sensitivity to cold stress in Ichang papeda.

## 3 Discussion

As specific transcription factors (TFs) in plants, WRKY proteins have been shown to participate in multiple plant stress resistances (Yang, Fang, et al. 2025) and various groups of family members were reported to play as activators or repressors in cold tolerance (Tang et al. 2022; Zhang et al. 2022; Guo et al. 2024; Bao et al. 2025). In this study, we presented multiple lines of evidence that support the role of CiWRKY27 as a novel regulator of cold tolerance in Ichang papeda. The expression of *CiWRKY27* demonstrated a substantial elevation under cold treatment, and heterologously overexpressed *CiWRKY27* significantly promoted the cold resistance of both lemon ( *C. limon* ) and tobacco (*N. nudicaulis*). These data collectively suggest that CiWRKY27 functions as a positive regulator in Ichang papeda cold resistance, which aligns with our previous studies showing that the silencing of *CiWRKY27* via VIGS markedly increased the sensitivity of Ichang papeda plants to cold stress (Qu et al. 2025). Given that CiWRKY27 is a functional transcriptional activator, it will be informative to identify and characterise its various direct targets to dissect the multifaceted functions of CiWRKY27. To elucidate the mechanisms underlying the CiWRKY27‐mediated transcriptional regulation in cold resistance, a combination of RNA‐seq and DAP‐seq was employed, which uncovered 717 direct CiWRKY27‐regulated target genes. Among them, a series of target genes were significantly upregulated by cold stress and have been demonstrated to be associated with abiotic stress. For instance, *PtrZAT12* of trifoliate orange has been shown to positively mediate cold resistance (Zhang, Xiao, et al. 2023) and *CiWRKY31* has been identified as a positive regulator of cold resistance (Qu et al. 2024). The *ATL31* and *PUB22* genes have also been demonstrated to function in various stresses (Ahn et al. 2022; Zhang, Tong, et al. 2023). This finding suggests that CiWRKY27 functions as a global regulator, directly regulating multiple aspects of pathways to resist cold stress.

Previous studies have highlighted the critical roles of glutathione metabolism and phenylpropanoid metabolic pathways in plants' cold stress response (Choudhury et al. 2016; Dong and Lin 2021). Based on this scenario, we screened the differentially expressed genes pertinent to ROS scavenging and lignin biosynthesis pathways, revealing that the expression levels of *CiGSTF6* and *CiCAD7* were tremendously repressed in the VIGS Ichang papeda lines, while predominantly elevated in OE lemons. The high similarity in up‐regulation between *CiGSTF6*, *CiCAD7* and CiWRKY27 under cold treatment suggests that *CiGSTF6* and *CiCAD7* might be integral to the response to cold stress. Integrative analyses confirmed that CiWRKY27 directly and specifically binds to the W‐box element within the promoters of these two genes and activates their expression. Enhanced lignification and antioxidant activity were observed in *CiWRKY27*‐overexpressing lemon plants subjected to cold stress. Moreover, silencing *CiGSTF6* and *CiCAD7* in Ichang papeda resulted in reduced cold tolerance. Hence, CiWRKY27 modulates lignin biosynthesis and ROS homeostasis, thereby alleviating cold stress injury. These results are reminiscent of reports on several key enzyme genes involved in lignin biosynthesis, including phenylalanine ammonia‐lyase (PAL), 4‐coumarate‐CoA ligase (4CL) and CAD, whose expressions were significantly induced by ROS (Liu et al. 2024), indicating a potential correlation between these two metabolic pathways. However, the precise molecular mechanism underlying this crosstalk remains to be fully elucidated. In tomato, SlGSTU43 interacts with SlCOMT2 to modulate lignin content and enhance salt tolerance (Yuan et al. 2024). Our study shifts the focus from interactions among functional proteins to firstly justifying the CiWRKY27‐*CiGSTF6* and CiWRKY27‐*CiCAD7* transcriptional regulatory modules. It implies that CiWRKY27 acts as a bridge mediating the crosstalk between ROS homeostasis and lignin synthesis, thereby providing compelling evidence to account for the variation in cold tolerance observed in the transgenic plants. It is noted that the expression levels of *CiWRKY27*, *CiGSTF6* and *CiCAD7* were significantly increased in both cold‐tolerant Ichang papeda and cold‐sensitive HB pummelo, implying that the regulatory role of *CiWRKY27* appears to be conserved across citrus species. However, the induction of these genes was markedly stronger and more sustained in the cold‐tolerant species (Figure S16). This greater expression in the tolerant genotype may facilitate a more robust activation of downstream defense pathways, thereby contributing to improved cold tolerance. Surprisingly, no difference in the promoter sequences of these genes was observed between Ichang papeda and HB pummelo (Figure S17), suggesting that divergent epigenetic modification or post‐transcriptional regulation may account for the difference in expression.

TF interaction plays a crucial role in plant responses to various abiotic stresses, resulting in altered protein abundance, transcript levels, and transcriptional activity (Jiang et al. 2020; Zhang, Zhu, et al. 2023; Liu et al. 2025). However, the interactions between WRKY and RAP2 transcription factors during cold stress remain poorly understood. In this study, we identified CiRAP2.7 as an interactor of CiWRKY27. Given that RAP2.7 interacts with WRKY27, we investigated whether RAP2.7 also influences the regulation of *CiCAD7*. A luciferase assay demonstrated that CiRAP2.7 not only activates *CiCAD7* alone but also collaborates with CiWRKY27 to augment its expression. To further elucidate the regulatory relationship between these two transcription factors (TFs), a series of verification experiments confirmed that CiRAP2.7 functions not only as an interactor but also as a target of CiWRKY27. Extensive research has demonstrated that AP2‐ERF genes play significant roles in responses to abiotic stress, with key transcription factors C‐REPEAT BINDING FACTORS (CBFs) being central to cold resistance mechanisms (Guo et al. 2018; Fu et al. 2025). Furthermore, numerous studies have explored the roles of *RAP2* genes in abiotic stress. For instance, members of the ERF‐VII family in *Arabidopsis*, including RAP2.2, RAP2.3, and RAP2.12, exhibit redundant functions in various stress responses (Papdi et al. 2015; Tang et al. 2020; Li et al. 2022). Furthermore, RAP2.6 has been shown to interact with CDK8 and SnRK2.6 to regulate the expression of *RD29A* and *COR15A*, thereby modulating ABA signalling during drought tolerance (Zhu et al. 2020). Nonetheless, research on RAP2.7 has been relatively sparse, only reporting that RAP2.7 in cotton may serve as a potential contributor to abiotic stress responses by modulating hormone‐associated biochemical pathways during the process of domestication based on the phylogenomic analysis (Peng et al. 2022). Our findings provide empirical evidence that CiRAP2.7 is induced by cold and functions as a key nuclear‐localised transcriptional activator. Additionally, we observed that CiRAP2.7 exerted a positive influence on the cold resistance of Ichang papeda. Hence, these results could advance our understanding of *RAP2* genes' functions in plants to resist cold stress, which holds significant potential for application in citrus molecular breeding programmes aimed at developing cold‐tolerant cultivars. Moreover, unlike the classical CBF‐dependent pathways that have been reported to be activated at an early stage of cold stress (Kim et al. 2024), the CiWRKY27‐*CiRAP2.7* module exhibited a late response to cold stress, implying that WRKY27 may regulate RAP2.7 to regulate target genes under such conditions via a CBF‐independent pathway. However, it is plausible that WRKY27 also participates in other CBF‐dependent regulatory pathways, and further detailed experiments are required to validate this hypothesis.

**FIGURE 10:**
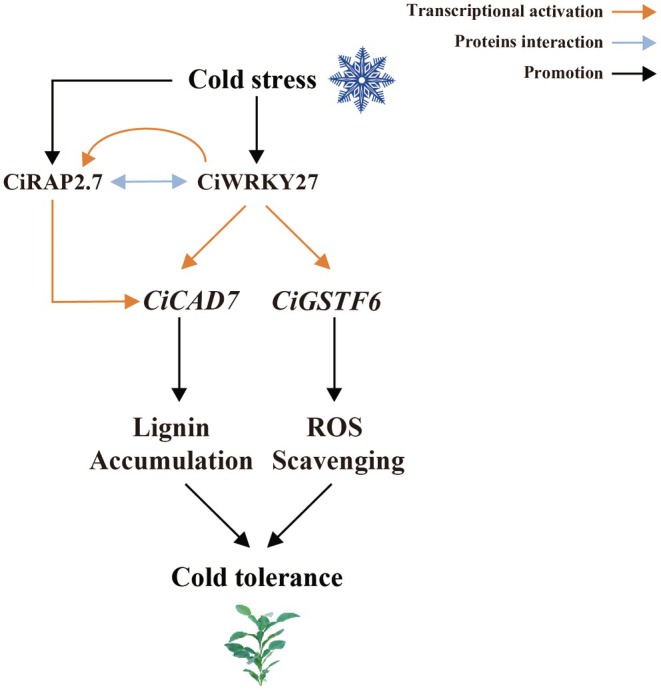
A proposed model illustrating the CiWRKY27‐mediated cold tolerance in Ichang papeda (*Citrus ichangensis*). Cold stress results in the activation of CiWRKY27, which serves as the transcriptional activator of *CiCAD7* and *CiGSTF6*. In addition, *CiRAP2.7* interacts with CiWRKY27, strengthening the transcriptional activation ability of the latter. Meanwhile, CiWRKY27 regulates *CiRAP2.7*, which in turn controls *CiCAD7*. As a result, CiWRKY27 and *CiRAP2.7* together regulate *CiCAD7* and *CiGSTF6* to promote lignin biosynthesis and ROS detoxification, leading to enhanced cold tolerance.

Based on our data, we propose a working model depicting a molecular mechanism of CiWRKY27 function in cold stress response in Ichang papeda (Figure 10). Cold stress results in activation of CiWRKY27, which functions to act as the upstream transcriptional activator of *CiCAD7* and *CiGSTF6*. On the other hand, *CiRAP2.7* interacts with CiWRKY27 and strengthens its transcriptional activation through an unknown mechanism. Meanwhile, CiWRKY27 transcriptionally regulates *CiRAP2.7*, which can in turn regulate *CiCAD7*. As a result, the molecular module CiWRKY27–CiRAP2.7 works synergistically to regulate *CiCAD7* and *CiGSTF6*, leading to elevated biosynthesis of lignin and promoted ROS detoxification for imparting cold tolerance. Taken together, our findings advance our understanding of the molecular mechanism involved in lignin accumulation and ROS elimination in plant response to cold stress, and provide valuable clues for exploring crucial pathways for manipulating cold tolerance in citrus.

## 4 Materials and Methods

### 4.1 Plant Materials, Growth Conditions and Cold Treatments

Seeds of Ichang papeda (*C. ichangensis*) harvested from the orchards of Huazhong Agriculture University were sown in the soil pots, which were then incubated at 25°C under a light/night cycle of 16/8 h. Three‐month‐old seedlings were placed in a growth chamber set at 4°C, and the leaves were sampled at 0, 6, 24, 72, 120 h. In addition, 3‐month‐old seedlings were treated with 10 mM of abscisic acid (ABA), brassinosteroid (BR), methyl jasmonate (MeJA) and salicylic acid (SA) under normal conditions. Leaves were collected at 0, 6, 12, 24, 48 h following the exogenous treatment to assess the expression of *CiWRKY27*. All samples were immediately frozen in liquid nitrogen and stored at −80°C for further analysis.

### 4.2 RT‐qPCR Analysis

Total RNA was extracted from the leaves, roots, stems, peels and pulp of Ichang papeda by a commercial RNA extraction kit (RN3302; Biotech Co. Ltd., Beijing, China). The RNA was subsequently reverse transcribed with HiScript III RT SuperMix (Vazyme, Nanjing, China). The RT‐qPCR was conducted using AceQ SYBR Green Master Mix (Vazyme, Nanjing, China) on an ABI7500 system (Applied Biosystems, Foster City, CA, USA), following the previously described method (Ming et al. 2020). Gene expression levels were determined following the $2^{-\Delta \Delta C_{t}}$ method (Livak and Schmittgen 2001), using *Actin* as an internal control. All primers used for this assay, unless otherwise stated, were listed in Table S1.

### 4.3 Subcellular Localisation Analysis

The coding sequences (CDS) of *CiWRKY27* and *CiCAD7* without the stop codons were amplified and subsequently cloned into the p101YFP (yellow fluorescent protein) vector, generating 35S: CiWRKY27‐YFP or 35S: CiRAP2.7‐YFP constructs. The fusion constructs or the pYFP101 control vector were co‐transformed with the nuclear marker (35S: VirD2NLS‐mCherry) into the *N. benthamiana* leaves. The YFP fluorescent signals emitted by the target proteins were observed using a confocal laser scanning microscope (Leica TCS SP8 DSL, Wetzlar, Germany).

### 4.4 Transcriptional Activation Assay

The complete CS of *CiWRKY27* and *CiRAP2.7* and their truncated fragments were separately fused to the pGBKT7 vector (Clontech, CA, USA). The recombinant fusion constructs were transferred into the yeast *AH109* strain (Takara, Shiga, Japan) using a yeast transformation kit (Clontech, USA). The pGBKT7‐53 + pGADT7‐T and pGBKT7 were used as the positive and negative controls. The transformants were selected on SD/−Trp and SD/−Trp/−Leu/−His medium without or with X‐α‐gal (5‐Bromo‐4‐chloro‐3‐indolyl‐α‐D‐galactoside, Coolaber, Beijing, China). In addition, transcriptional activation activity was assessed using an in vivo dual‐luciferase (LUC) reporter assay. The full‐length CDS of *CiWRKY27* and *CiRAP2.7* was cloned into the pBD vector to generate the GAL4 pBD, serving as the effector. The 5 × GAL4‐LUC vector was used as the reporter. The assays in *N. benthamiana* leaves were performed as described previously (Khan et al. 2021).

### 4.5 Vector Construction and Plant Transformation

To generate overexpressing lines, the complete CDS of *CiWRKY27* was cloned into the pGWB411 vector using Gateway recombination technology (Invitrogen, USA). Subsequently, the resultant construct was introduced into tobacco (*N. nudicaulis*) and lemon ( *C. limon* ) through agrobacterium‐mediated transformation and cultivated on MS (for tobacco) or MT medium (for lemons) supplemented with 50 μg/mL kanamycin according to Fu et al. (2011). The expression levels of transgenes in the positive transgenic lines were assessed by RT‐qPCR. The T_2_ generation of transgenic tobacco lines and the vegetatively propagated plants of lemon were used for further analysis.

### 4.6 Virus‐Induced Gene Silencing (VIGS)

The partial cDNA fragments of *CiWRKY27* (303 bp), *CiRAP2.7* (300 bp), *CiGSTF6* (300 bp) and *CiCAD7* (300 bp) were amplified and inserted into the pTRV2 (Tobacco Rattle Virus) vector. The recombinant constructs, pTRV1 and pTRV2, were individually transformed into the *A. tumefaciens* strain GV3101. The bacterial suspension containing pTRV1 was mixed with either pTRV2 or pTRV2‐target genes in equal volumes and transformed into 30‐d‐old Ichang papeda seedlings according to previously described protocols (Dai et al. 2018). One month later, the leaves were harvested and used for genomic PCR and RT‐qPCR for confirming potential positive transgenic plants. The 3‐month‐old VIGS plants were subjected to cold treatment and other analyses.

### 4.7 Cold Treatments of Transgenic Plants

For cold treatment, 3‐week‐old tobacco plants were subjected to 4 h at 4°C, followed by 8 h at −2°C, prior to recovery for 24 h at room temperature. The survival rates were then assessed. As for lemon, both wild type (WT) and transgenic lines were exposed to −2°C for 4 h, followed by −4°C treatment. In addition, the VIGS plants were exposed to −4°C. The cold treatment was terminated when the phenotypic difference between lemon transgenic lines and WT or between the VIGS plants and TRV:00 control was conspicuous. The leaves were used for chlorophyll fluorescence imaging and then harvested for subsequent analysis.

### 4.8 Physiological Measurement and Histochemical Staining

The commercially detection kits (Nanjing Jiancheng Bioengineering Institute, China) were employed to evaluate the concentrations of total protein (A045‐2‐2) and malondialdehyde (MDA, A003‐1) in the analysed samples, following the manufacturer's guidelines. Electrolyte leakage (EL) was measured as previously described (Dahro et al. 2016). Chlorophyll fluorescence measurements were obtained using an IMAGING‐PAM chlorophyll fluorometer (Walz, Germany), and the *Fv/Fm* ratio was calculated with Imaging Win software. The accumulation of H_2_O_2_ and $O_{2}^{.-}$ was assessed by histochemical staining with 3, 3′‐diaminobenzidine (DAB) and nitro blue tetrazolium (NBT), respectively according to Huang et al. (2013).

### 4.9 Quantification of Lignin Content and CAD Activity

Stems from wild type (WT) and *CiWRKY27*‐overexpressing lines were fixed in FAA fixative and embedded in molten paraffin wax. The embedded blocks were mounted on a microtome holder (Leica SM2000R, Wetzlar, Germany), trimmed and sectioned at 15 μm thickness. For lignin detection, the sections were treated with 100 μL acidified solution to saturate tissues, followed immediately by adding an equal volume of phloroglucinol‐HCl staining solution. After 5 min of incubation, the samples were imaged by light microscopy (3D HISTECH PANNORAMIC MIDI, Budapest, Hungary). The measurements of CAD activity and lignin content were conducted based on the enzyme‐linked immunosorbent assay (ELISA) method using a specific detection kit (Mlbio, Shanghai, China) following the manufacturer's instructions.

### 4.10 DAP‐Seq and Data Analysis

DAP‐seq was performed based on previously established methods (O'Malley et al. 2016; Bartlett et al. 2017). The CiWRKY27 target protein was recombined to the promegaG184A vector containing a HaloTag sequence through the TNT SP6 High‐Yield Wheat Germ Protein Expression System (Promega, USA). The HaloTag Beads (Promega, USA) were used to purify the recombinant CiWRKY27‐HaloTag, followed by the incubation of CiWRKY27‐HaloTag or HaloTag beads (Input, negative control) mixture with the Ichang papeda genomic DNA library. The construction of the DAP‐seq library and sequencing was performed using the Nova Seq 6000 platform. The clean reads were mapped to the reference genome of *C. ichangensis* using BWA software (Li and Durbin 2009; Liu, Wang, et al. 2022). DeepTools software was used to analyse the distribution of reads within 2 kb upstream and downstream of the gene transcription start site (TSS) or gene ontology (GO) region and plot the reads density distribution (Ramírez et al. 2014). ChIPseeker R package was used to analyse the distribution of reads over different genes' functional elements across the genome and illustrated in a Venn diagram. The identification of DAP‐seq peaks was completed by MACS with the default parameters (*Q*‐value < 0.001, fold change > 2; Zhang et al. 2008). Motifs were predicted by MEME (https://meme‐suite.org/meme/). The enriched peaks were visualised by IGV (Robinson et al. 2011). GO annotation and Kyoto Encyclopaedia of Genes and Genomes (KEGG) enrichment analysis (Ashburner et al. 2000; Kanehisa and Goto 2000) were completed using the Dr. Tom Multi‐omics Data Mining System (https://biosys.bgi.com).

### 4.11 RNA‐Seq and Data Analysis

RNA‐seq of TRV:00 control and VIGS plants, three biological replicates for each, was performed according to Zhang et al. (2021). In brief, total RNA was isolated from the leaves sampled from the plants before and after cold treatment by an RNA extraction kit (RN38, Aidlab Biotech Co. Ltd., Beijing, China). The construction of cDNA library and sequencing on the DNBSEQ platform were performed by BGI (Shenzhen, China). Raw reads were filtered by SOAPnuke (Chen et al. 2017) and then mapped to the reference genome of *C. ichangensis* using HISAT (Kim et al. 2015) and Bowtie2. Differentially expressed genes (DEGs) were selected by DEseq2 (Love et al. 2014) based on the following criteria: *Q*‐value < 0.05 and fold change ≥ 2, as illustrated on a volcano plot. The GO annotation and KEGG enrichment analysis were conducted as mentioned above. Cluster analysis was completed by R package pheatmap.

### 4.12 Yeast Hybrid Assays (Y1H and Y2H)

For yeast one‐hybrid (Y1H) assay, the promoter sequences of target genes were amplified and fused to the pAbAi vector to generate the baits. The full‐length CDS of *CiWRKY27* and *RAP2.7* was inserted into the pGADT7 vector to get the preys. The baits and prey were co‐transformed into *Y1HGold* yeast cells in light of instructions provided by Matchmaker Gold Yeast One‐Hybrid Library Screening System (Clontech, USA), which were selected on the SD/‐Ura/−Leu medium with or without aureobasidin A (AbA) (Coolaber, Beijing, China). pGADT7‐P53 + p53‐AbAi and pGADT7‐AD + bait were used as the positive and negative control, respectively.

For Yeast two‐hybrid (Y2H) assay, the full‐length CDS of *CiWRKY27* and truncated segments (ΔC: 1–618 bp; TF domain: 619–798 bp; and ΔN: 799–1206 bp) were separately fused to the pGBKT7 vector, which served as the bait and was transformed with pGADT7 to test self‐activation. pGBKT7‐53 + pGADT7‐T and pGBKT7‐lam + pGADT7‐T were used as the positive and negative controls, respectively. Fragments without self‐activation were chosen to be the baits for Y2H library screening. The library screening was conducted according to the Nuclear Mating System instruction manual (Oebiotech, Shanghai, China). In brief, the *Y2HGold* yeast strain containing baits and Y187 library yeast strain containing preys were mixed together and cultured at low speed. Then, transformants were selected on SD/−Trp/−Leu and SD/−Trp/−Leu/‐His/−Ade with or without X‐α‐gal (Coolaber, Beijing, China) medium. The positive clones were picked for further sequencing and point‐to‐point validation.

### 4.13 Electrophoretic Mobility Shift Assay (EMSA)

The complete open reading frame (ORF) of CiWRKY27 was ligated to the pHMGWA vector (HIS‐tag) and induced at 37°C for 5 h, then purified using Ni‐NTA agarose (Qiagen, Hilden, Germany). The probes (Table S2) containing original or mutated W‐box motifs were synthesised and labeled by AuGCT Biological Technology (Wuhan, China), using unlabeled probes as DNA competitors. The electrophoretic mobility shift assay (EMSA) was performed following the procedures of the Chemiluminescent EMSA kit (Beyotime, Shanghai, China).

### 4.14 Dual Luciferase (LUC) Assay

The CDS of *CiWRKY27* or *CiRAP2.7* was integrated into the pGreenII 62‐SK vector, serving as the effectors. The promoter fragments of *CiGSTF6*, *CiCAD7* and *CiRAP2.7* containing W‐box were amplified and fused with the pGreenII 0800‐LUC vector to function as reporters. All constructs were introduced into the *Agrobacterium* strain *GV3101* harbouring the *pSoup* plasmid. The effector and reporter, along with p19, were then transiently infiltrated into *N. benthamiana* leaves at a ratio of 7:5:3 and cultured in the dark for 3 days, followed by fluorescence imaging on the Vivo Plant Imaging System (Night Shade LB985, Berthold, Germany). The activities of firefly LUC and *Renilla* (REN) were measured using a Dual‐Luciferase Reporter Assay System Kit (Promega, Madison, WI, USA) by Infinite 200 Pro microplate reader (Tecan, Mannedorf, Switzerland). Three biological replicates were conducted for each sample.

### 4.15 Bimolecular Fluorescence Complementation Assay

The bimolecular fluorescence complementation (BiFC) assay was conducted according to Zhang, Zhu, et al. (2023). The CDS of *CiWRKY27* and *CiRAP2.7* were amplified and fused to the C and N terminal, respectively, of yellow fluorescent protein (YFP) in the L101 vector. The resulting constructs nYFP‐CiRAP2.7 and cYFP‐CiWRKY27 were then introduced into *Agrobacterium* strain *GV3101* and transiently expressed in the leaves of 28‐day‐old *N. benthamiana* plants, using nYFP‐AtZIP63 + cYFP‐AtZIP63 and nYFP + cYFP as the positive and negative controls, respectively. The YFP signals were visualised using confocal laser scanning microscopy (Leica TCS‐SP8, Wetzlar, Germany).

### 4.16 LUC Complementation Imaging (LCI) Assay

The LCI assay was carried out to verify the interaction between CiWRKY27 and CiRAP2.7 proteins. The full‐length CiRAP2.7, lacking a stop codon, was cloned into the JW771 vector containing the N‐terminal LUC (nLUC), while CiWRKY27 was fused to the JW772 vector with the C‐terminal LUC (cLUC). Both recombinant proteins and empty vectors (nLUC and cLUC) were transformed into *Agrobacterium* strain *GV3101* and subsequently co‐infiltrated into *N. benthamiana* leaves in equal proportions. LUC fluorescence in the infiltrated leaves was detected using the Vivo Plant Imaging System (Night Shade LB985, Berthold, Germany).

### 4.17 Pull‐Down Assay

The coding sequence of CiRAP2.7 was amplified and integrated into the pGEX‐6P‐1‐GST vector (GE Healthcare, USA) to generate the GST‐CiRAP2.7 fusion protein. Both GST‐CiRAP2.7 and HIS‐CiWRKY27 (same as that of EMSA) were expressed in *Rosetta* (DE3) cells (TransGen Biotech, China) and subsequently purified using GST‐tag resin (Beyotime, Shanghai, China) and Ni‐NTA agarose (Qiagen, Hilden, Germany) following the manufacturer's manual. For the pull‐down assay, GST and GST‐RAP2.7 attached to the GST‐tag resin were incubated with HIS‐CiWRKY27 in GST pull‐down binding buffer (50 mM Tris–HCl, 200 mM NaCl, 1 mM EDTA, 1% NP‐40, 1 mM DTT and 10 mM MgCl_2_, pH 8.0). After 4 h of gentle rotation at 4°C, the mixture was washed with GST pull‐down washing buffer (50 mM Tris–HCl, 50 mM GSH, 400 mM NaCl, 1 mM EDTA and 1 mM DTT, pH 8.0). The eluted proteins were then analysed using immunoblotting with anti‐GST and anti‐HIS antibodies (Beyotime, Shanghai, China).

### 4.18 Statistical Analysis

The data, represented as means ± standard deviation (SD) from three replicates, were analysed using SPSS software (IBM, SPSS 22). A one‐way ANOVA was performed followed by the LSD test, taking significance at levels of *p* < 0.05 (*), *p* < 0.01 (**), or *p* < 0.001 (***).

### 4.19 Accession Numbers

Sequence data from this study can be found in *C. ichangensis* reference genome within Citrus Pan‐genome to Breeding Database (CPBD; http://citrus.hzau.edu.cn/) under the following accession numbers: *CiWRKY27* (Ci055870), *CiGSTF6* (Ci047590), *CiCAD7* (Ci024420), and *CiRAP2.7* (Ci224130). Abbreviations were listed in Table S5.

## Author Contributions

J.Q., C.L. and J.‐H.L. conceived the research idea. J.Q. and P.X. performed the experiments. Y.W. provided assistance for bioinformatics analysis. T.F. and H.C. participated in material acquisition. J.Q. prepared the initial draft of the manuscript. J.‐H.L. finalised the writing and editing of the final manuscript.

## Conflicts of Interest

The authors declare no conflicts of interest.

## Supporting information


**Figure S1:** Expression of *CiWRKY27* under exogenous hormone treatments.
**Figure S2:** CiWRKY27 acted as a cold‐inducible transcription factor.
**Figure S3:** Genetic transformation of lemon with overexpression of *CiWRKY27* and RT‐qPCR analysis of gene expression levels.
**Figure S4:** RT‐qPCR identification of transgenic tobacco (*N. tabacum*) plants overexpressing *CiWRKY27*.
**Figure S5:** Overexpression of *CiWRKY27* elevated cold tolerance in transgenic tobacco (*N. tabacum*).
**Figure S6:** The size and distribution of binding sites from DAP‐seq.
**Figure S7:** Cold‐induced enhancement of CAD enzyme activity and lignin content in Ichang papeda.
**Figure S8:** RT‐qPCR identification of *CiCAD7*‐VIGS plants.
**Figure S9:** RT‐qPCR identification of *CiGSTF6*‐VIGS plants.
**Figure S10:** Assessment of self‐activation and cytotoxicity for the bait in yeast two‐hybrid (Y2H) library screening.
**Figure S11:** PCR analysis of yeast colonies involved in yeast two‐hybrid (Y2H) library screening.
**Figure S12:** Analysis of *cis*‐acting motifs in the promoter of *CiWRKY27* and *CiRAP2.7*.
**Figure S13:** The expression levels of *CiRAP2.7* in the *CiWRKY27*‐VIGS lines using RT‐qPCR.
**Figure S14:** CiRAP2.7 acted as a cold‐inducible transcription factor.
**Figure S15:** RT‐qPCR identification of *CiRAP2.7*‐VIGS plants.
**Figure S16:** Expression dynamic of *CiWRKY27*, *CiGSTF6* and *CiCAD7* in cold‐tolerant and cold‐sensitive citrus species.
**Figure S17:** Compared analysis of *CgWRKY27* and *CiWRKY27* promoters.
**Table S1:** List of primers used in this study.
**Table S2:** List of probes used in the EMSA assay.
**Table S3:** The detail information of RNA‐seq analysis.
**Table S4:** The detail information of DAP‐seq analysis.
**Table S5:** The list of abbreviation.

## Data Availability

The data that support the findings in this study are available from the corresponding author upon reasonable request.
