# Supplementary material for: WRKY27‐RAP2.7 Regulatory Module Promotes Cold Tolerance via Modulation of Lignin Biosynthesis and Redox Homeostasis by Regulating Cinnamyl Alcohol Dehydrogenase 7 and Glutathione S‐Transferase F6
Source: Plant Biotechnol J. 2025 Nov 17;24(4):2021–39. doi: 10.1111/pbi.70439 (PMC13140723; doi:10.1111/pbi.70439)
Supplement: Supplementary file 1 — Figure S1: Expression of CiWRKY27 under exogenous hormone treatments. Figure S2: CiWRKY27 acted as a cold‐inducible transcription factor. Figure S3: Genetic transformation of lemon with overexpression of CiWRKY27 and RT‐qPCR analysis of gene expression levels. Figure S4: RT‐qPCR identification of transgenic tobacco (N. tabacum) plants overexpressing CiWRKY27. Figure S5: Overexpression of CiWRKY27 elevated cold tolerance in transgenic tobacco (N. tabacum). Figure S6: The size and distribution of binding sites from DAP‐seq. Figure S7: Cold‐induced enhancement of CAD enzyme activity and lignin content in Ichang papeda. Figure S8: RT‐qPCR identification of CiCAD7‐VIGS plants. Figure S9: RT‐qPCR identification of CiGSTF6‐VIGS plants. Figure S10: Assessment of self‐activation and cytotoxicity for the bait in yeast two‐hybrid (Y2H) library screening. Figure S11: PCR analysis of yeast colonies involved in yeast two‐hybrid (Y2H) library screening. Figure S12: Analysis of cis‐acting motifs in the promoter of CiWRKY27 and CiRAP2.7. Figure S13: The expression levels of CiRAP2.7 in the CiWRKY27‐VIGS lines using RT‐qPCR. Figure S14: CiRAP2.7 acted as a cold‐inducible transcription factor. Figure S15: RT‐qPCR identification of CiRAP2.7‐VIGS plants. Figure S16: Expression dynamic of CiWRKY27, CiGSTF6 and CiCAD7 in cold‐tolerant and cold‐sensitive citrus species. Figure S17: Compared analysis of CgWRKY27 and CiWRKY27 promoters. Table S1: List of primers used in this study. Table S2: List of probes used in the EMSA assay. Table S3: The detail information of RNA‐seq analysis. Table S4: The detail information of DAP‐seq analysis. Table S5: The list of abbreviation. [file PBI-24-2021-s001.docx]

Supplementary figures

**WRKY27-RAP2.7 regulatory module promotes cold tolerance via modulation of lignin biosynthesis and redox homeostasis by regulating *CINNAMYL ALCOHOL DEHYDROGENASE 7* and *GLUTATHIONE S-TRANSFERASE F6***

Jing Qu^1^, Peng Xiao^1^, Yilei Wang^1^, Tian Fang^1^, Haowei Chen^1^, Chunlong Li^1^, Ji-Hong Liu^1, 2^*

**Author affiliations**

^1^National Key Laboratory for Germplasm Innovation & Utilization of Horticultural Crops, College of Horticulture and Forestry Sciences, Huazhong Agricultural University, Wuhan 430070, China

^2^Hubei Hongshan Laboratory, Wuhan 430070, China.

***Correspondence**

Ji-Hong Liu ([liujihong@mail.hzau.edu.cn](mailto:liujihong@mail.hzau.edu.cn))

**Supporting Information**

Figure S1. Expression of *CiWRKY27* under exogenous hormone treatments.

Figure S2. CiWRKY27 acted as a cold-inducible transcription factor*.*

Figure S3. Genetic transformation of lemon with overexpression of *CiWRKY27* and RT-qPCR analysis of gene expression levels.

Figure S4. RT-qPCR identification of transgenic tobacco (*N. tabacum*) plants overexpressing *CiWRKY27*.

Figure S5. Overexpression of *CiWRKY27* elevated cold tolerance in transgenic tobacco (*N. tabacum*).

Figure S6. The size and distribution of binding sites from DAP-seq.

Figure S7. Cold-induced enhancement of CAD enzyme activity and lignin content in Ichang papeda.

Figure S8. RT-qPCR identification of *CiCAD7*-VIGS plants.

Figure S9. RT-qPCR identification of *CiGSTF6*-VIGS plants.

Figure S10. Assessment of self-activation and cytotoxicity for the bait in yeast two-hybrid (Y2H) library screening.

Figure S11. PCR analysis of yeast colonies involved in yeast two-hybrid (Y2H) library screening.

Figure S12. Analysis of *cis*-acting motifs in the promoter of *CiWRKY27* and *CiRAP2.7*.

Figure S13. The expression levels of *CiRAP2.7* in the CiWRKY27-VIGS lines using RT-qPCR.

Figure S14. CiRAP2.7 acted as a cold-inducible transcription factor*.*

Figure S15. RT-qPCR identification of *CiRAP2.7*-VIGS plants.

Figure S16. Expression dynamic of *CiWRKY27*, *CiGSTF6*, and *CiCAD7* in cold-tolerant and cold-sensitive citrus species.

Figure S17. Compared analysis of *CgWRKY27* and *CiWRKY27* promoters.

Table S1. List of primers used in this study.

Table S2. List of probes used in the EMSA assay.

Table S3. The detail information of RNA-seq analysis.

Table S4. The detail information of DAP-seq analysis.

Table S5. The list of abbreviation.


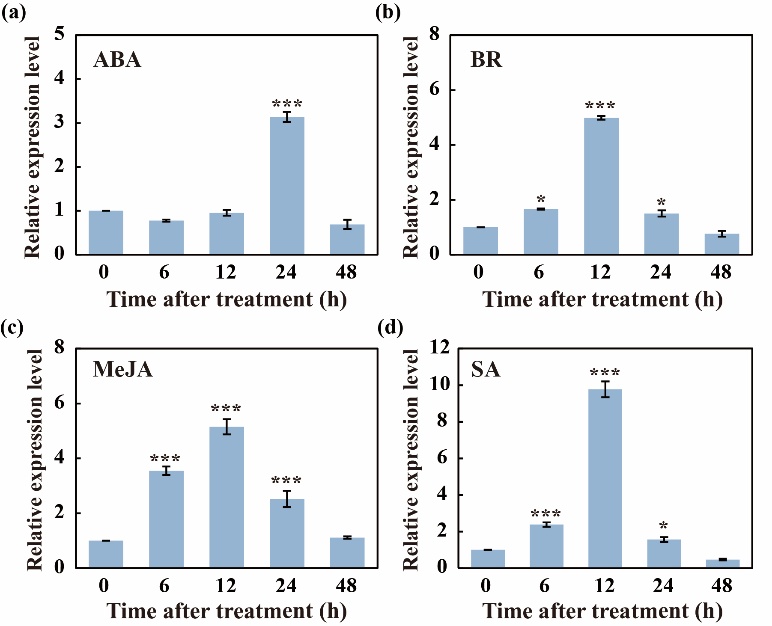


**Figure S1.** Expression of *CiWRKY27* under exogenous hormone treatments. Relative expression levels of *CiWRKY27* in response to ABA (a), BR (b), MeJA (c), and SA (d) treatment at normal condition, as measured by RT-qPCR. Asterisks indicate significant differences compared with the level at 0 h under the same growth condition, **P* < 0.05; ****P* < 0.001.


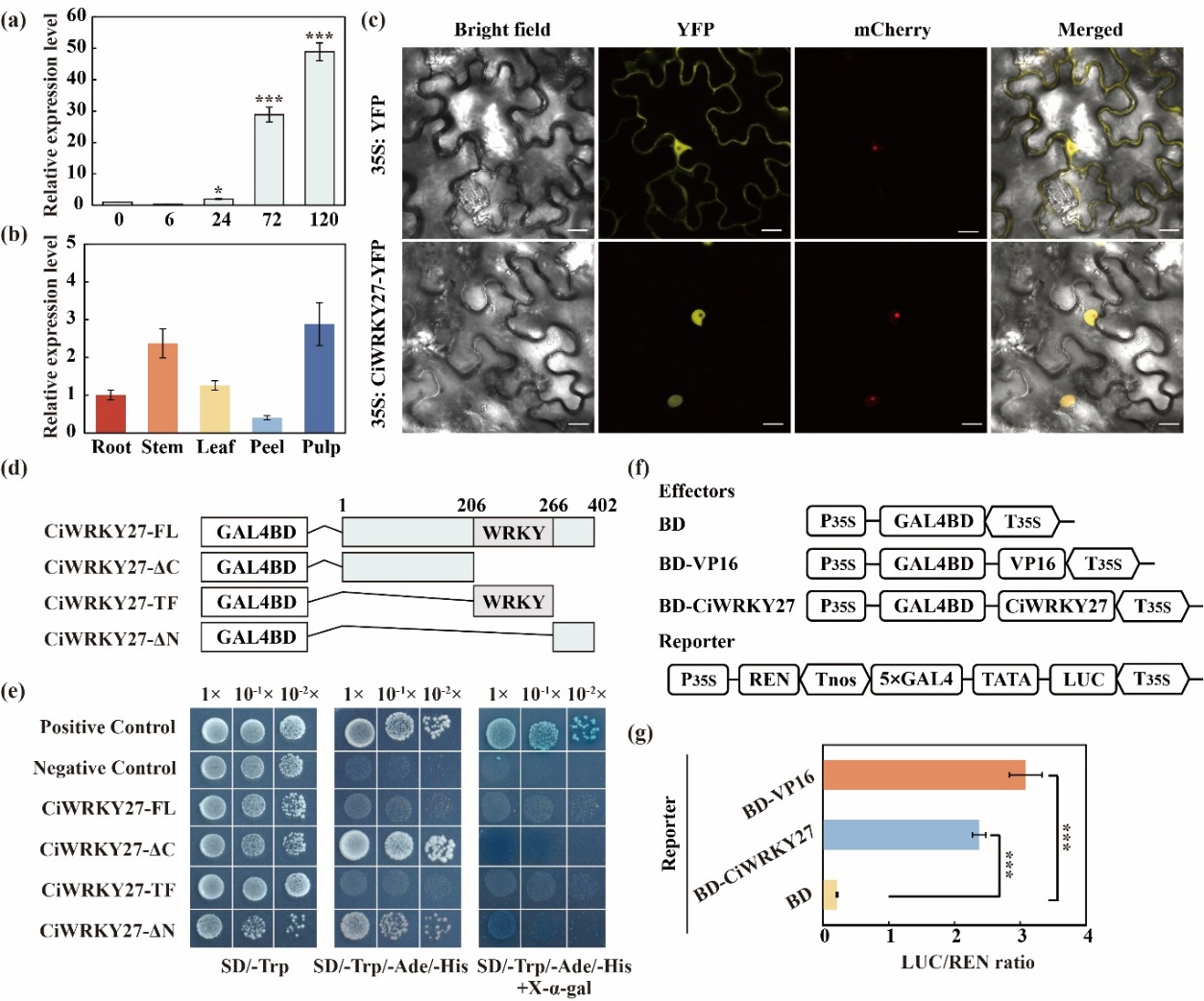


**Figure S2.** CiWRKY27 acted as a cold-inducible transcription factor*.* (a-b) Expression pattern of *CiWRKY27* in Ichang papeda (*C. ichangensis*) leaves at designed time points of cold treatment (4 °C) (a) and in various tissues under normal growth conditions (b), as determined by RT-qPCR. (c) Subcellular localization of CiWRKY27 using mCherry as a nuclear marker for co-localization. YFP, yellow fluorescent protein. mCherry, red fluorescent protein. Bars, 25 µm. (d) Schematic diagram of constructs used for transcriptional activity assays. The full-length (CiWRKY27-FL) and three truncated coding sequence (CDS) of CiWRKY27 were fused with the GAL4 DNA-binding domain in pGBKT7 and transformed into *AH109* yeast cells. CiWRKY27-ΔC and CiWRKY27-ΔN refer to the C and N-terminal truncation, respectively. CiWRKY27-TF (transcription factor) represent the region containing the WRKY domain. The number above the bars indicate the positions of amino acid residues. (e) Growth of yeast cells co-transformed with various vectors, at different dilutions, on the selective medium added with or without X-α-Gal (5-Bromo-4-chloro-3-indolyl-α-D-galactoside). (f) Schematic diagram of effectors and reporter constructs used for transcriptional activity assays. BD and VP16 were used as negative and positive control, respectively. (g) Reporter activity, as measured by LUC/REN ratios, in *Nicotiana benthamiana* leaves infected with different constructs. LUC, firefly luciferase; REN, *Renilla* luciferase. Error bars indicate ± SD (n = 3). Asterisks indicate significant differences compared with the level at 0 h (a) or between groups under the same growth condition (g), **P* < 0.05; ****P* < 0.001.


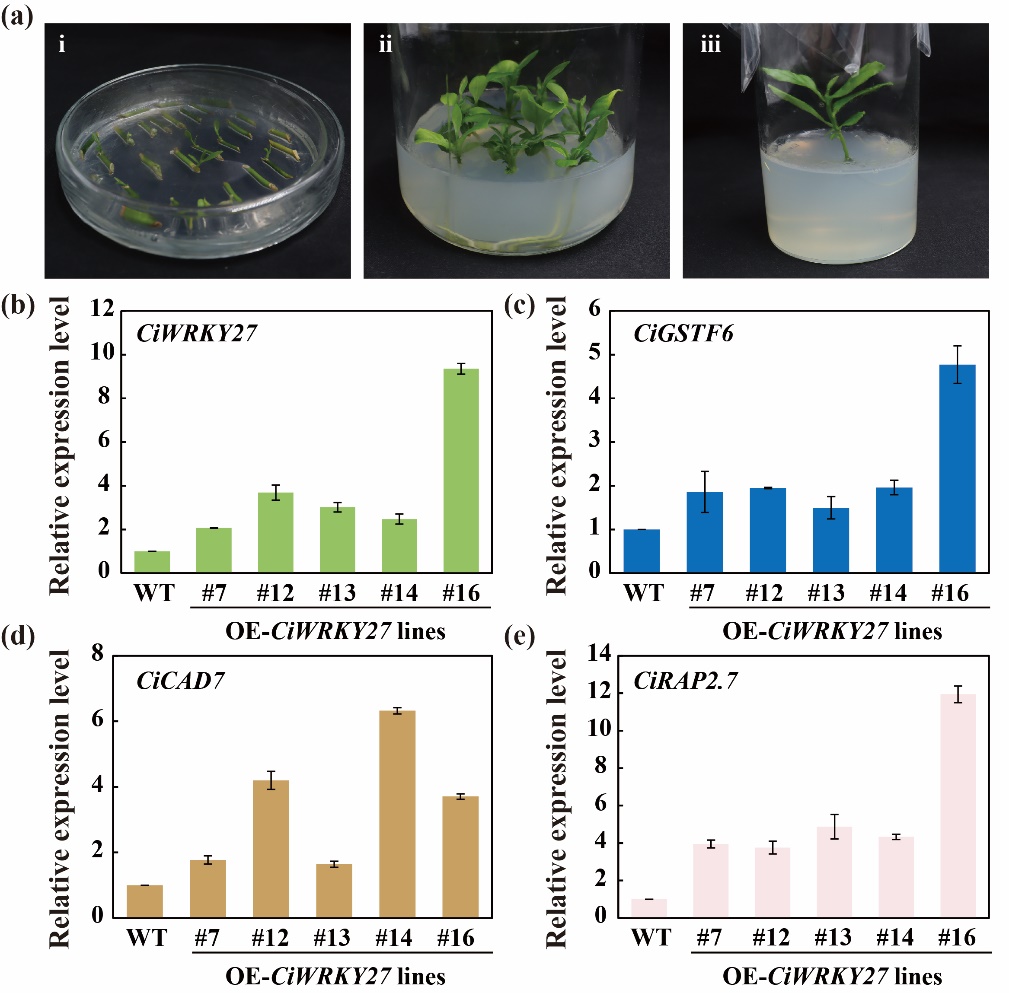


**Figure S3.** Genetic transformation of lemon with overexpression of *CiWRKY27* and RT-qPCR analysis of gene expression levels. (a) The transformation and regeneration of lemon: (a)-i the growth of shoot segments; (a)-ii The Elongation and multiplication of shoots; (a)-iii Development of rooted plants. (b-e) RT-qPCR was performed to detect the expression of *CiWRKY27* (b) and its targeted genes (*CiGSTF6*, *CiCAD7*, and *CiRAP2.7*) in overexpression lemon plants.


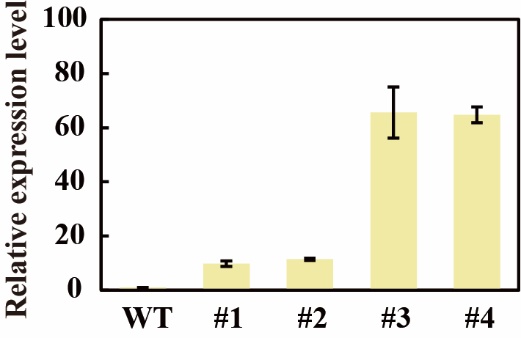


**Figure S4.** RT-qPCR identification of transgenic tobacco (*N. tabacum*) plants overexpressing *CiWRKY27*. Analysis of *CiWRKY27* expression in tobacco wild type (WT) and overexpressing lines by RT-qPCR. *Ubiqutin* was used as an internal control. Error bars represent means ± SD of three replicates.

**
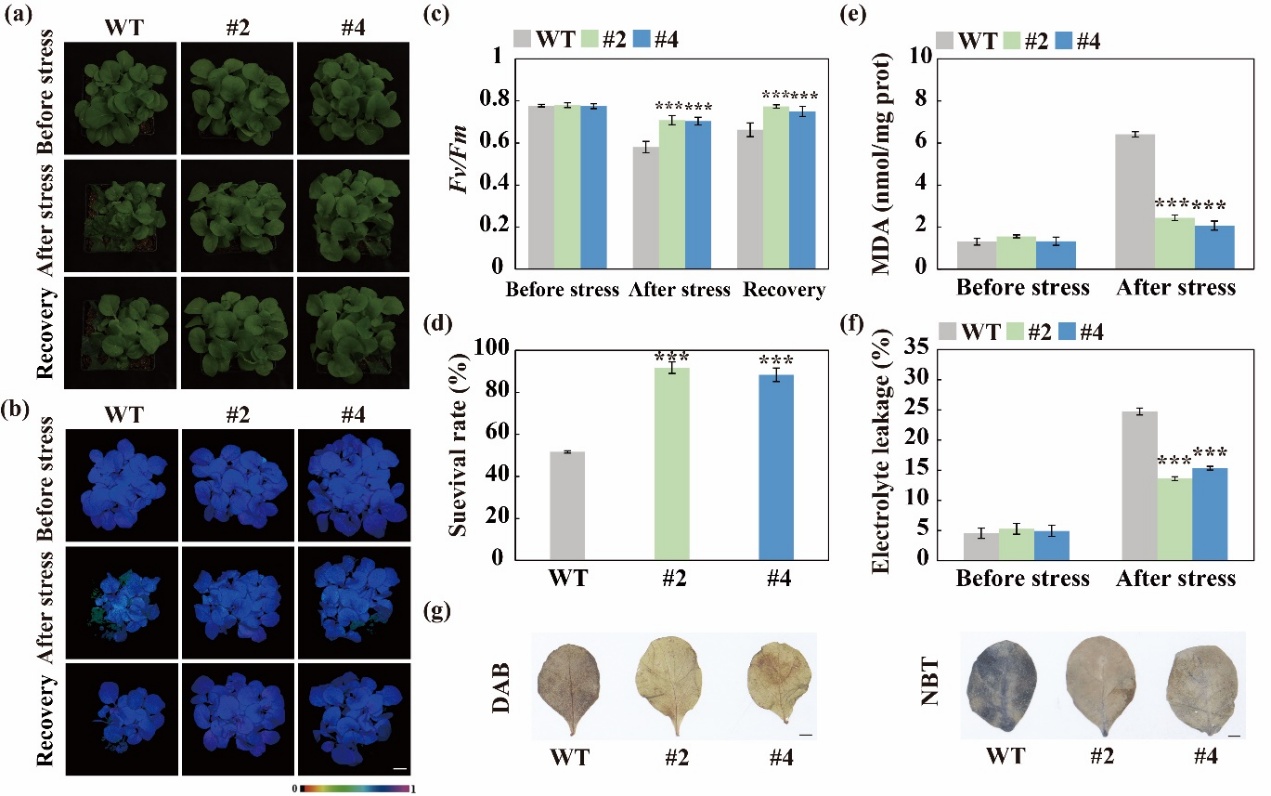
**

**Figure S5.** Overexpression of *CiWRKY27* elevated cold tolerance in transgenic tobacco (*N. tabacum*). (a) Phenotypes of wild-type (WT) and transgenic tobacco plants (#2 and #4) before and after cold treatment (4 h at 4 °C, then 8 h at -2 °C), followed by recovery for 1 d at ambient environment. Scale bars = 2 cm. (b-f) Chlorophyll fluorescence imaging (b), *Fv*/*Fm* ratios (c), survival rate (d), MDA content (e), and EL (f) of WT and transgenic tobacco plants measured before and after cold treatment. The false color scale between 0 and 1 is shown under the imaging. (g) Histochemical staining with 3,3’-diaminobenzidine (DAB, left) and nitro bule tetrazolium (NBT, right) of leaves sampled from the tested plants after the cold treatment. Scale bars = 1 cm. ANOVA method was conducted for significant analysis and marked by asterisks (****P* < 0.001).


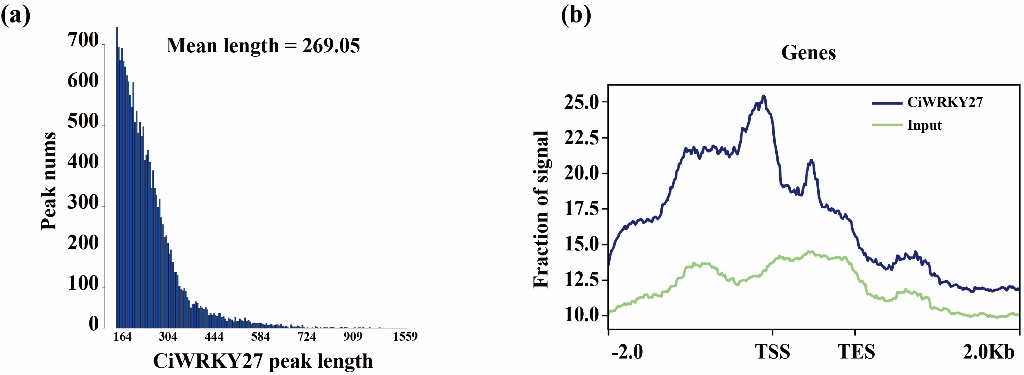


**Figure S6.** The size and distribution of binding sites from DAP-seq. (a) Distribution of CiWRKY27-binding peak length. (b) Metaplot of CiWRKY27 binding sites in different regions. TSS, transcription start sites. TES, transcription end site.


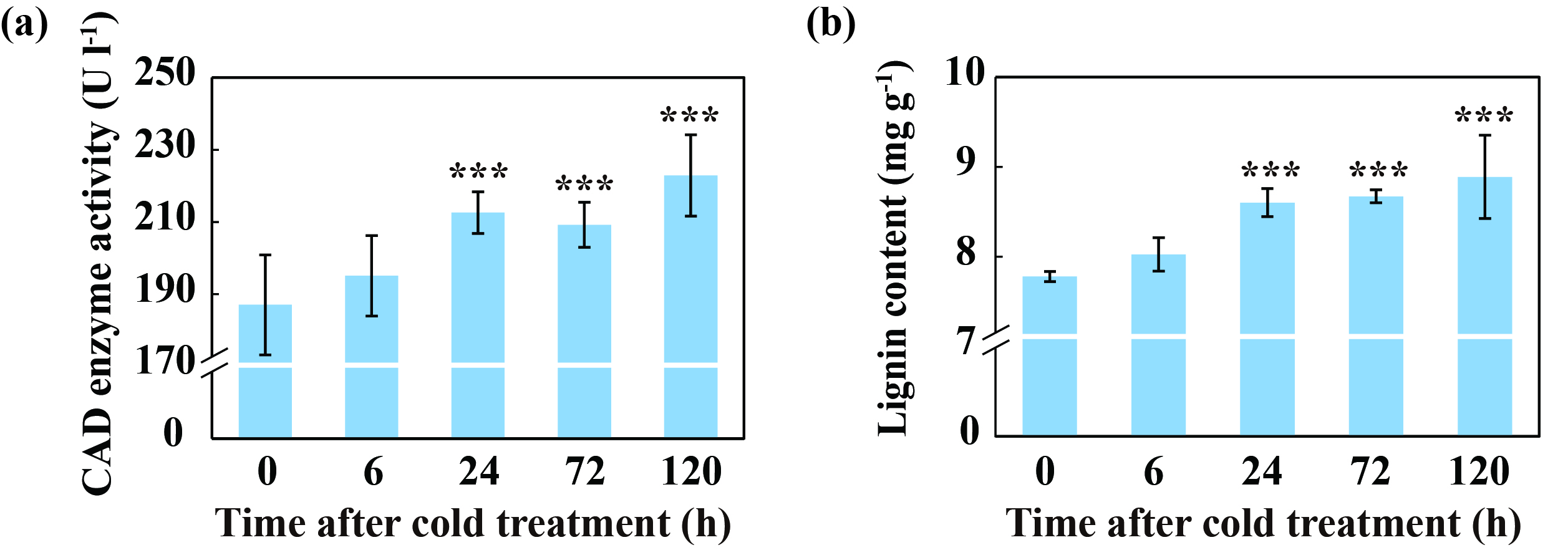


**Figure S7.** Cold-induced enhancement of CAD enzyme activity and lignin content in Ichang papeda. The activity of CAD activity (a) and lignin content (b) in wild-type Ichang papeda subjected to cold treatment for designated time were measured. Error bars indicate ± SD (n = 3). Asterisks indicate significant differences compared with the level at 0 h, ****P* < 0.001.

**
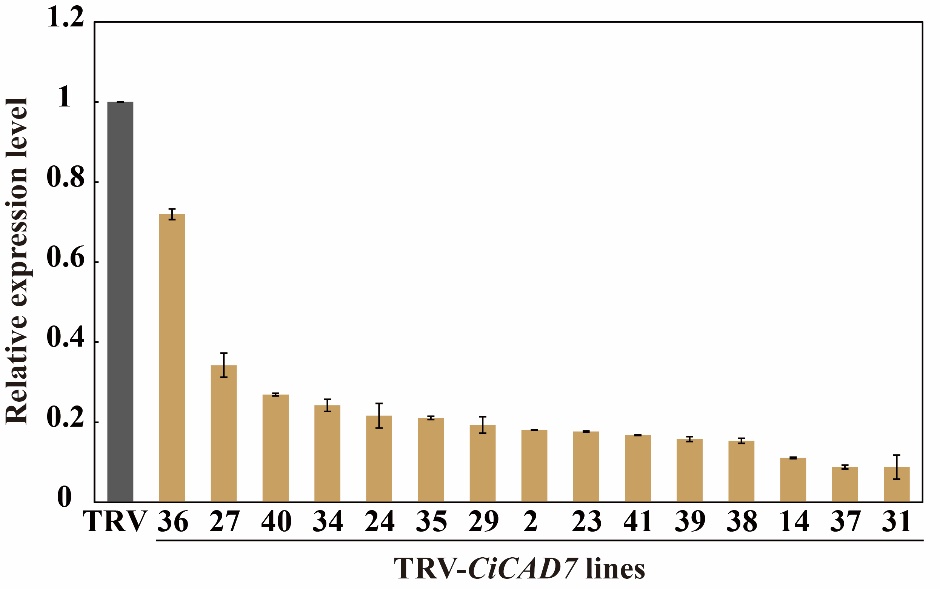
**

**Figure S8.** RT-qPCR identification of *CiCAD7*-VIGS plants. The expression level of *CiCAD7* was analyzed in TRV control (TRV) and VIGS plants (TRV-*CiCAD7*) by RT-qPCR. *Actin* was used as an internal control. Error bars represent means ± SD of three replicates.


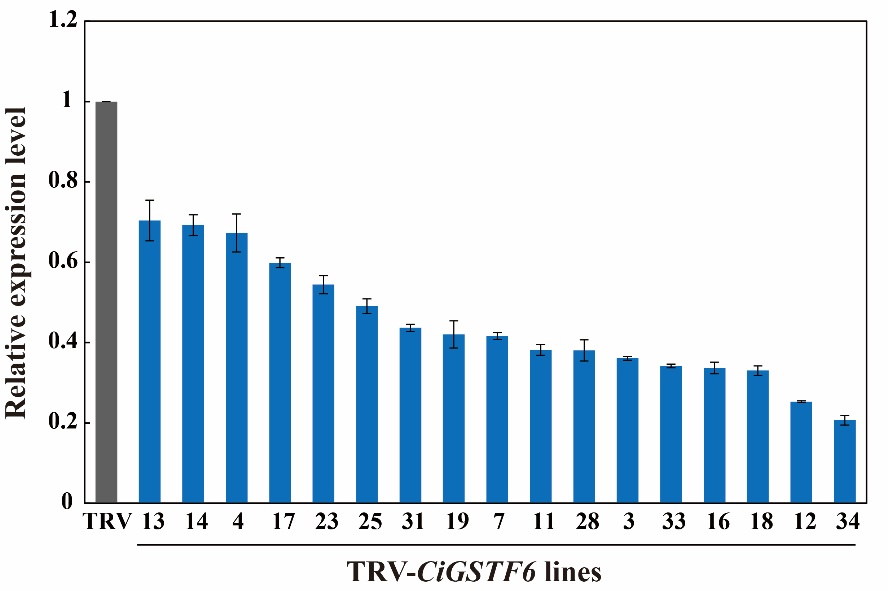


**Figure S9.** RT-qPCR identification of *CiGSTF6*-VIGS plants. The expression level of *CiGSTF6* was detected in TRV control (TRV) and VIGS plants (TRV- *CiGSTF6*) by RT-qPCR. *Actin* was used as an internal control. Error bars represent means ± SD of three replicates.


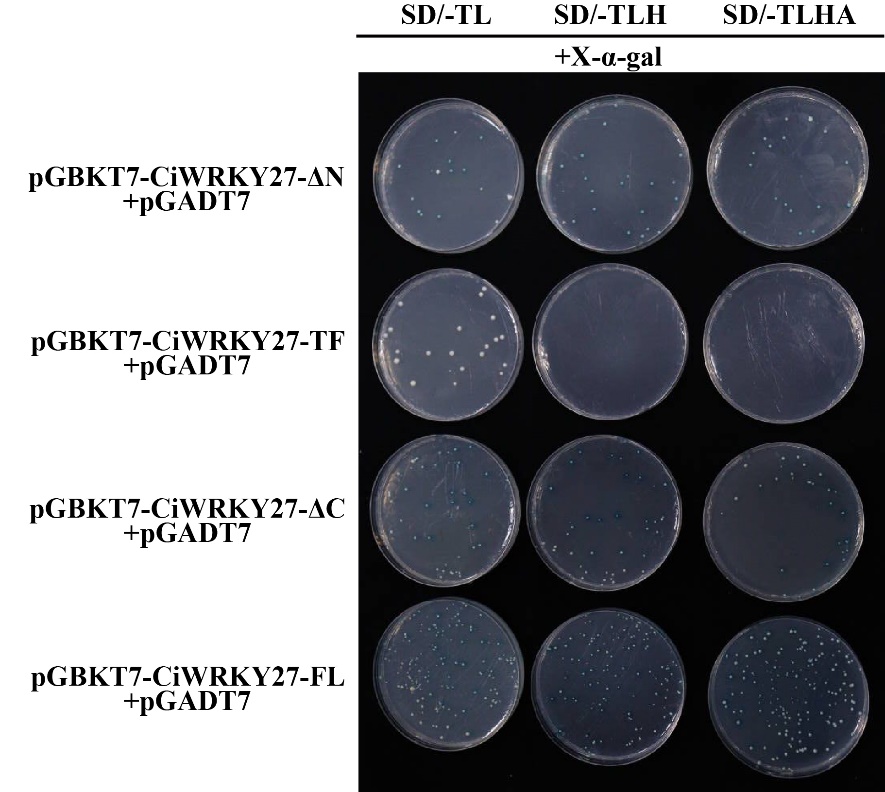


**Figure S10.** Assessment of self-activation and cytotoxicity for the bait in yeast two-hybrid (Y2H) library screening. Transformants containing various baits (pGBKT7-CiWRKY27-FL/ΔC/TF/ΔN) and pGADT7 were applied on synthetic dropout (SD) minimal base supplemented with X-α-gal. The media were deficient in different combinations of tryptophan (T, Trp), Leucin (L, Leu), Histidine (H, His), and adenine (A, Ade).

**
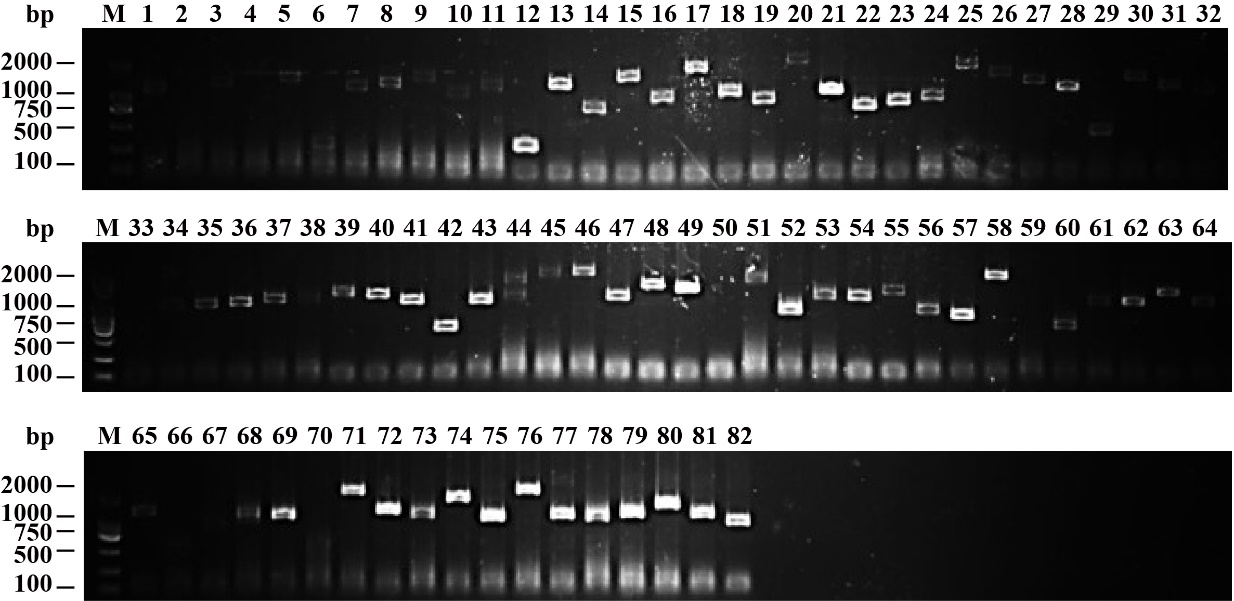
**

**Figure S11.** PCR analysis of yeast colonies involved in yeast two-hybrid (Y2H) library screening. The yeast colonies were randomly peaked from the selected medium (SD/-TLHA) and employed by PCR to identify the potential CiWRKY27 interact protein.


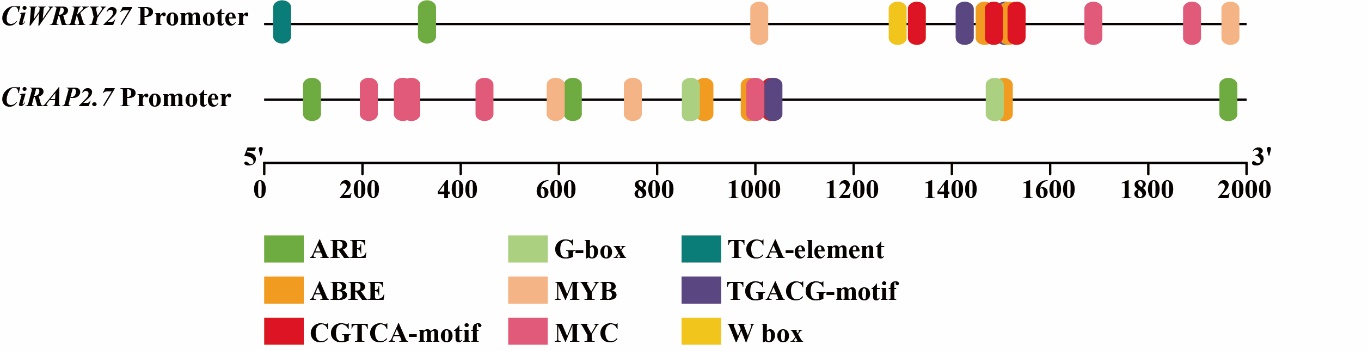


**Figure S12.** Analysis of cis-acting motifs in the promoter of *CiWRKY27* and *CiRAP2.7*. Presence of typical stress-associated *cis*-acting elements in promoters of *CiWRKY27* and *CiRAP2.7* within 2 kb.

**
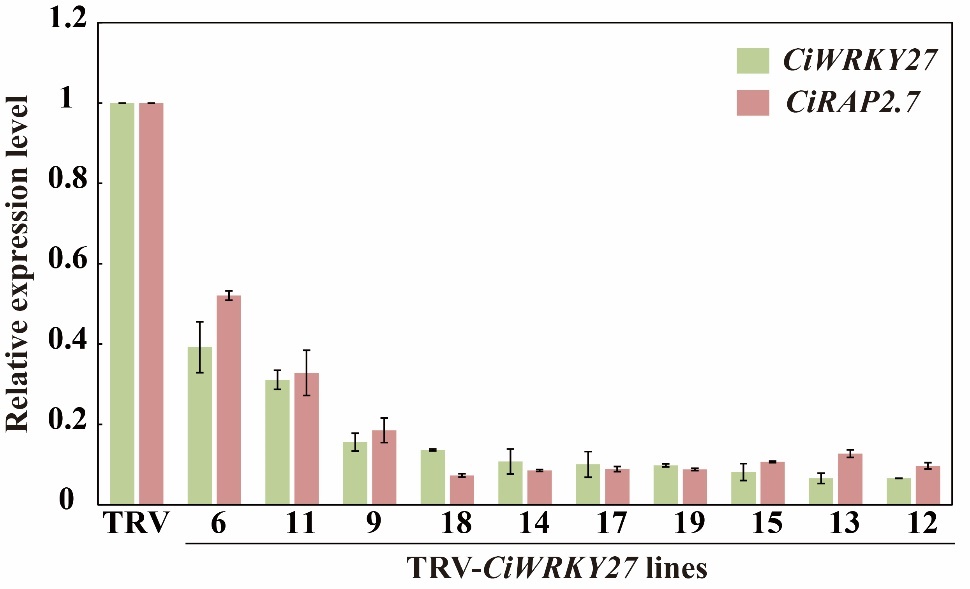
**

**Figure S13.** The expression levels of *CiRAP2.7* in the *CiWRKY27*-VIGS lines using RT-qPCR. The expression level of *CiRAP2.7* in *CiWRKY27*-VIGS plants (TRV-*CiWRKY27*) was analyzed by RT-qPCR. *Actin* was used as an internal control. Error bars represent means ± SD of three replicates.


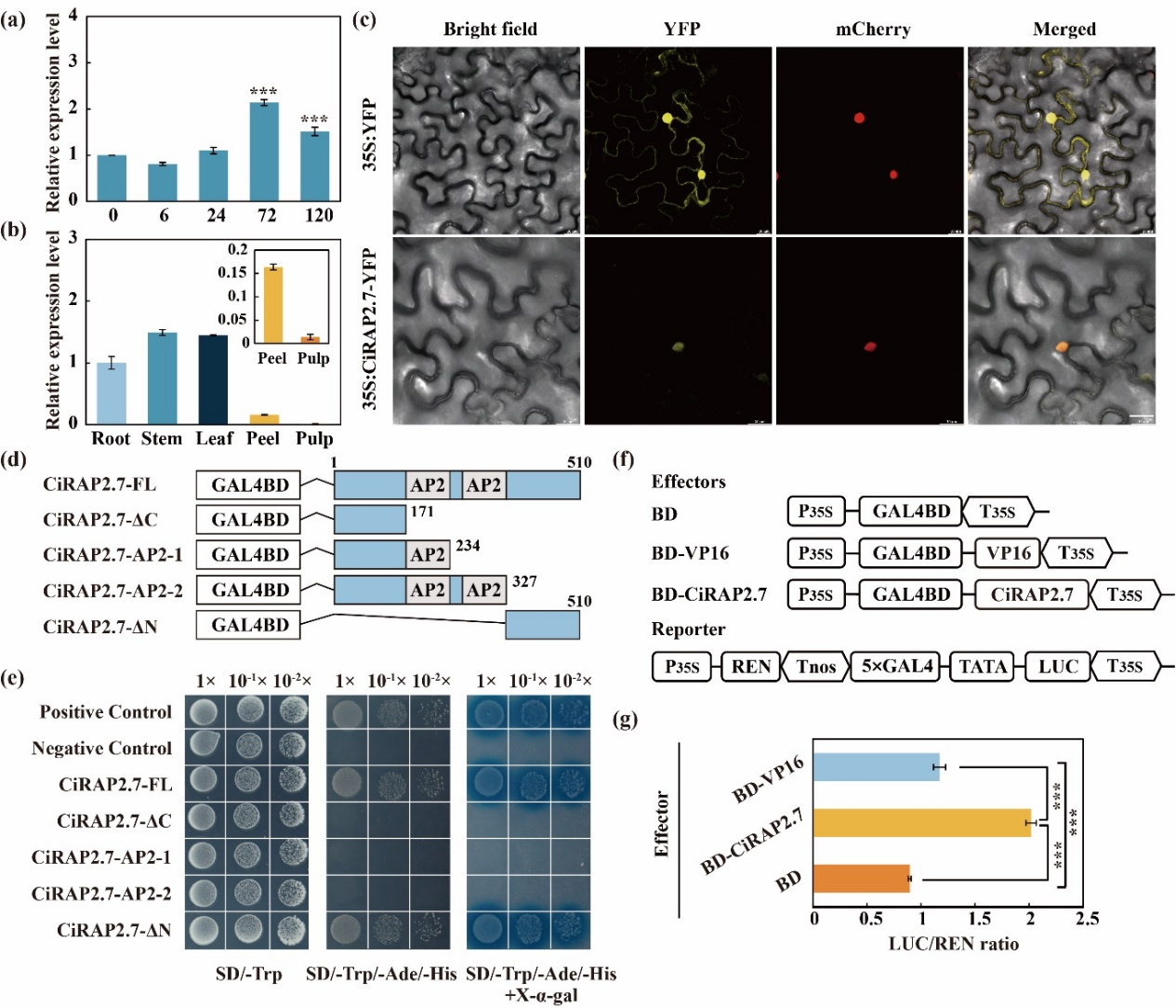


**Figure S14.** CiRAP2.7 acted as a cold-inducible transcription factor. (a-b) Expression pattern of *CiRAP2.7* in Ichang papeda at designed time points of cold treatment (4 °C) (a) and in various tissues under normal growth conditions (b), as determined by RT-qPCR. (c) Subcellular localization of CiRAP2.7 using mCherry as a nuclear marker for co-localization. YFP, yellow fluorescent protein. mCherry, red fluorescent protein. Bars, 20 µm. (d) Schematic diagram of constructs used for transcriptional activity assays. The full-length (CiRAP2.7-FL) and four truncated coding sequence (CDS) of CiRAP2.7 were fused with the GAL4 DNA-binding domain in pGBKT7 and transformed into *AH109* yeast cells. CiRAP2.7-ΔC and CiRAP2.7-ΔN refer to the C and N-terminal truncation, respectively. CiRAP2.7-AP2-1 and CiRAP2.7-AP2-2 represent the region containing the AP2 domain. The number above the bars indicate the positions of amino acid residues. (e) Growth of yeast cells co-transformed with various vectors, at different dilutions, on the selective medium added with or without X-α-Gal. (f) Schematic diagram of effectors and reporter constructs used for transcriptional activity assays. BD and VP16 were used as negative and positive control, respectively. (g) Reporter activity, as measured by LUC/REN ratios, in *N. benthamiana* leaves infected with different constructs. LUC, firefly luciferase; REN, *Renilla* luciferase. Error bars indicate ± SD (n = 3). Asterisks indicate significant differences compared with the level at 0 h (a) or between groups under the same growth condition (g), ****P* < 0.001.

**
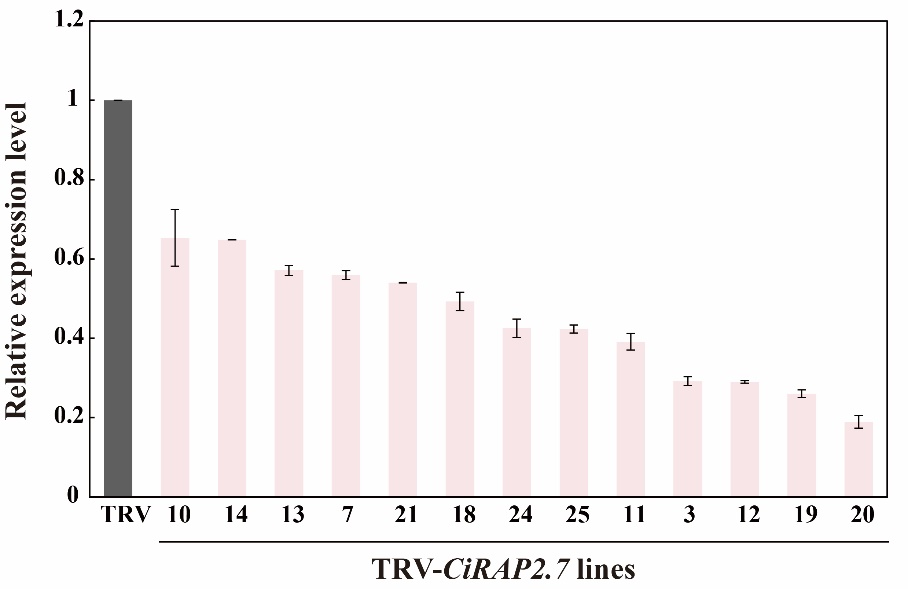
**

**Figure S15.** RT-qPCR identification of *CiRAP2.7*-VIGS plants. The expression level of *CiRAP2.7* was detected in TRV control (TRV) and VIGS plants (TRV-*CiRAP2.7*) by RT-qPCR. *Actin* was used as an internal control. Error bars represent means ± SD of three replicates.


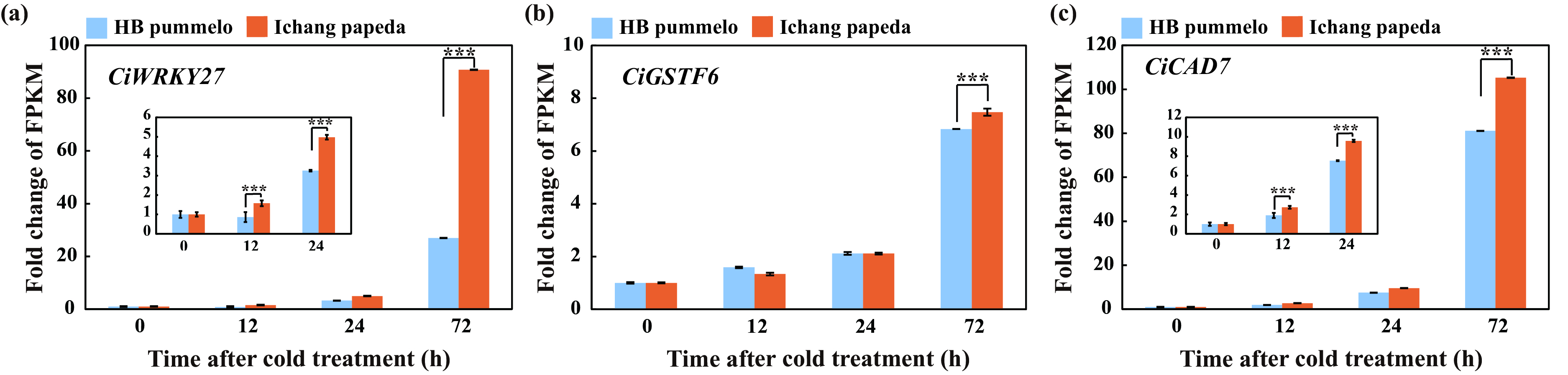


**Figure S16.** Expression dynamic of *CiWRKY27*, *CiGSTF6*, and *CiCAD7* in cold-tolerant and cold-sensitive citrus species. The expression dynamics of *CiWRKY27* (a), *CiGSTF6* (b), and *CiCAD7* (c) were compared between cold-tolerant Ichang papeda and cold-sensitive HB pummelo, based on the fold changes in their FPKM values. Error bars represent means ± SD of three replicates. Asterisks indicate significant differences compared with the level at different group, ****P* < 0.001.


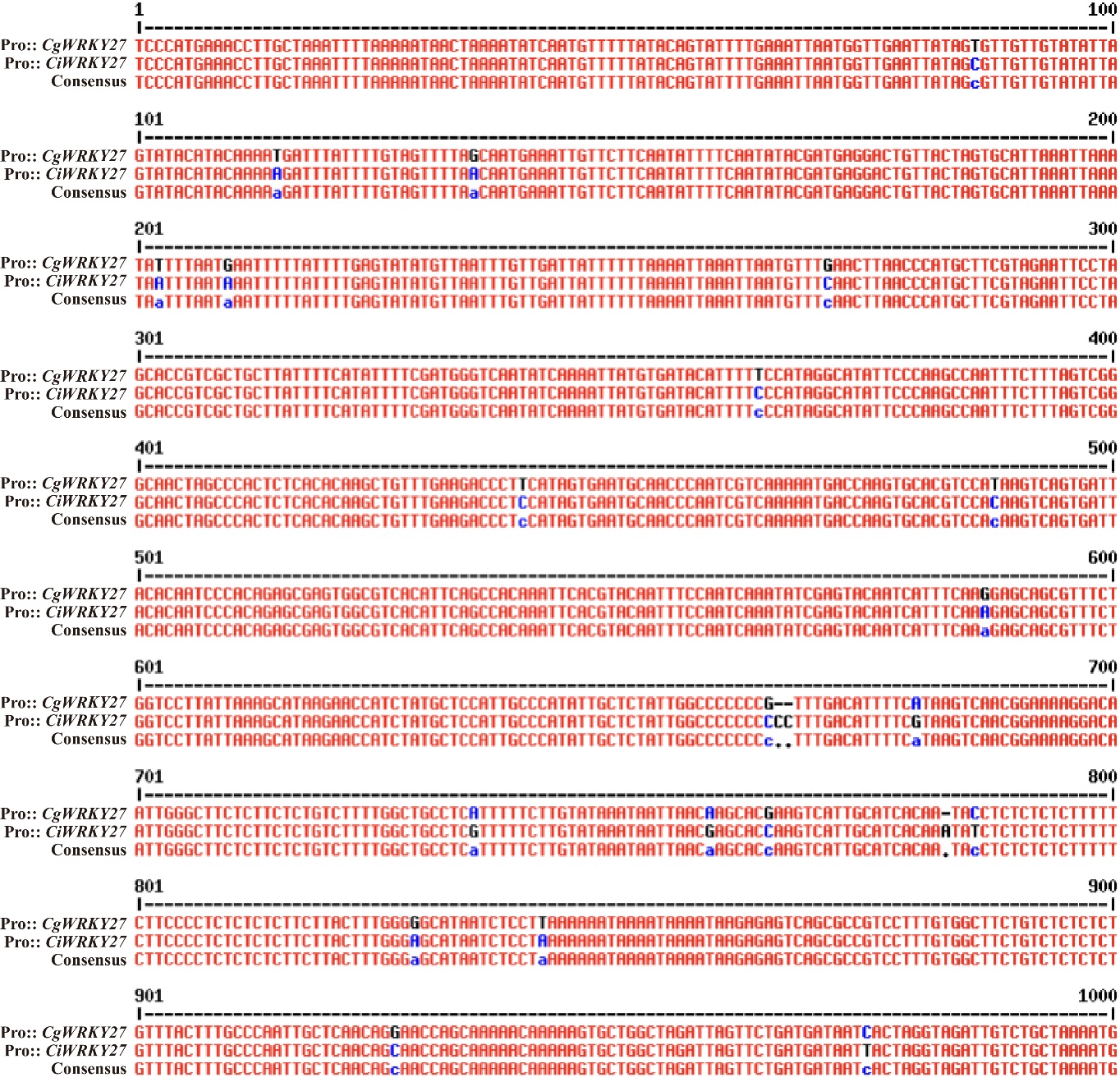


**Figure S17.** Compared analysis of *CgWRKY27* and *CiWRKY27* promoters. The promoters of *CgWRKY27* and *CiWRKY27* within 1 kb were aligned.

| **Table S1. List of primers used in this study** | |
| --- | --- |
| qPCR-*CiWRKY27*-F | AGCGGTGCCGCCGCCGCCGCCGATG |
| qPCR-*CiWRKY27*-R | AGTTGTTGGGGCCGACGGGGACTGAC |
| qPCR-*CiGSTF6*-F | CCATTCGGTCAAGTTCCGGT |
| qPCR-*CiGSTF6*-R | TACCAATAACGGGGCGATGG |
| qPCR-*CiCAD7*-F | CAGTTCACCCTCTAATGCCGT |
| qPCR-*CiCAD7*-R | TGCCACCAACTATCTTCCTCC |
| qPCR-*CiRAP2.7*-F | AGTTCAGGGGTGTTGATGCC |
| qPCR-*CiRAP2.7*-R | GGCCGCATTTGTGTAGTGTC |
| *CiWRKY27*-F | GCCCAATTGCTCAACAGCAACCAGC |
| *CiWRKY27-R* | AGCTCAGGAGACTATCAGCCGTGGT |
| *CiCAD7*-F | ATGGGGCAAGCACCGGAGCAAGAG |
| *CiCAD7*-R | TTAAGGGGTGGACTTCATTGTGTTTGC |
| *CiGSTF6*-F | ATGGCAGGCATCAAGGTCCACG |
| *CiGSTF6*-F | ATGGCAGGCATCAAGGTCCACG |
| *CiRAP2.7*-F | ATGTTGGATCTCAATCTAAAT |
| *CiRAP2.7*-R | CTATGGTGGTGCCTGCGGCAG |
| YFP-CiWRKY27-F (*EcoR*I) | ATGGGATCTACTAGTGAATTCATGGGTGAGAAATTT |
| YFP-CiWRKY27-R (*Bam*HⅠ) | GGGGGTACCGTCGACGGATCCGCCGTGGTTGTCGCC |
| YFP-CiRAP2.7-F (*EcoR*I) | GGATCTACTAGTGAATTCATGTTGGATCTCAAT |
| YFP-CiRAP2.7-R (*Bam*HⅠ) | GGTACCGTCGACGGATCCTTGGGTATCTCCAACTTTGG |
| pDONR221-*CiWRKY27*-F | GGACAAGTTTGTACAAAAAAGCAGGCTCCATGGGTGAGAAATTT |
| pDONR221-*CiWRKY27*-R | GGACCACTTTGTACAAGAAAGCTGGGTCTCAGCCGTGGTTGTC |
| pTRV2-*CiWRKY27*-F (*Bam*HI) | AGAAGGCCTCCATGGGGATCCATGGGTGAGAAATTT |
| pTRV2-*CiWRKY27*-R (*Sma*I) | TGTCTTCGGGACATGCCCGGGAGCAGAGCTTGGAGT |
| pTRV2-*CiCAD7*-F (*Bam*HI) | AGAAGGCCTCCATGGGGATCCATGGGGCAAGCACCG |
| pTRV2-*CiCAD7*-R (*Sma*I) | TGTCTTCGGGACATGCCCGGGCACCATGCATCCTAC |
| pTRV2-*CiRAP2.7*-F (*Bam*HI) | AGAAGGCCTCCATGGGGATCCATGTTGGATCTCAAT |
| pTRV2-*CiRAP2.7*-R (*Sma*I) | TGTCTTCGGGACATGCCCGGGCTATGGTGGTGCCTG |
| pBD-CiWRKY27-F (*Age*I) | AGTTGACTGTATCGCCGACCGGTATGGGTGAGAAATTT |
| pBD-CiWRKY27-R (*Stu*I) | TAATGAAACCAGAGTTAAAGGCCTTCAGCCGTGGTTGTC |
| pBD-CiRAP2.7-F (*Age*I) | AGTTGACTGTATCGCCGACCGGTATGTTGGATCTCAAT |
| pBD-CiRAP2.7-R (*Stu*I) | TAATGAAACCAGAGTTAAAGGCCTCTATGGTGGTGCCTG |
| pGBKT7-CiWRKY27-F (*EcoR*I) | ATGGCCATGGAGGCCGAATTCATGGGTGAGAAATTT |
| pGBKT7-CiWRKY27-R (*Sal*I) | TGCGGCCGCTGCAGGTCGACGCCGTGGTTGTCGCC |
| pGBKT7-CiWRKY27*-*ΔC-F (*EcoR*I) | ATGGCCATGGAGGCCGAATTCATGGGTGAGAAATTT |
| pGBKT7-CiWRKY27*-*ΔC-R (*Sal*I) | TGCGGCCGCTGCAGGTCGACGACTTGGCACACCAT |
| pGBKT7-CiWRKY27*-*TF-F (*EcoR*I) | ATGGCCATGGAGGCCGAATTCACGGAAGAGAAGCTT |
| pGBKT7-CiWRKY27*-*TF-R (*Sal*I) | TGCGGCCGCTGCAGGTCGACCCTGTGAGTTGGACG |
| pGBKT7-CiWRKY27*-*ΔN-F (*EcoR*I) | ATGGCCATGGAGGCCGAATTCAACTCGCTTGCTGGC |
| pGBKT7-CiWRKY27*-*ΔN-R (*Sal*I) | TGCGGCCGCTGCAGGTCGACGCCGTGGTTGTCGCC |
| pGBKT7-CiRAP2.7-FL-F (*EcoR*I) | ATGGCCATGGAGGCCGAATTCATGTTGGATCTCAAT |
| pGBKT7-CiRAP2.7-FL-R (*Sal*I) | TGCGGCCGCTGCAGGTCGACCTATGGTGGTGCCTG |
| pGBKT7-CiRAP2.7-ΔC-F (*EcoR*I) | ATGGCCATGGAGGCCGAATTCATGTTGGATCTCAAT |
| pGBKT7-CiRAP2.7-ΔC-R (*Sal*I) | TGCGGCCGCTGCAGGTCGACAGAGCTCCTTGACCT |
| pGBKT7-CiRAP2.7-AP-1-F (*EcoR*I) | ATGGCCATGGAGGCCGAATTCATGTTGGATCTCAAT |
| pGBKT7-CiRAP2.7-AP-1-R (*Sal*I) | TGCGGCCGCTGCAGGTCGACGTCCTCGTAATCAGC |
| pGBKT7-CiRAP2.7-AP-2-F (*EcoR*I) | ATGGCCATGGAGGCCGAATTCATGTTGGATCTCAAT |
| pGBKT7-CiRAP2.7-AP-2-R (*Sal*I) | TGCGGCCGCTGCAGGTCGACCCCTTCATATGTGCT |
| pGBKT7-CiRAP2.7-ΔN-F (*EcoR*I) | ATGGCCATGGAGGCCGAATTCGAGATGATTACTGAG |
| pGBKT7-CiRAP2.7-ΔN-R (*Sal*I) | TGCGGCCGCTGCAGGTCGACCTATGGTGGTGCCTG |
| *ProCiCAD7*-F | CATACCAAGTACTGG |
| *ProCiCAD7-*R | GGAGTGGCTATTCAT |
| *ProCiGSTF6-*F | GCTCGTTCGCAATAC |
| *ProCiGSTF6-*R | TTGGCGCCCACCTTT |
| *ProCiRAP2.7-*F | CCCTCTACGAGGCTCACTA |
| *ProCiRAP2.7-*R | CCAATTCCGACGGCTGAGAT |
| *pAbAi-proCiCAD7*-F (*Kpn*Ⅰ) | CTTGAATTCGAGCTCGGTACCCATACCAAGTACTGG |
| *pAbAi-proCiCAD7*-R (*Xho*Ⅰ) | ATACAGAGCACATGCCTCGAGGGAGTGGCTATTCAT |
| *pAbAi-proCiGSTF6-*F (*Kpn*Ⅰ) | CTTGAATTCGAGCTCGGTACCGCTCGTTCGCAATAC |
| *pAbAi-proCiGSTF6-*R (*Xho*Ⅰ) | ATACAGAGCACATGCCTCGAGTTGGCGCCCACCTTT |
| *pAbAi-proRAP2.7-*F1*-*F (*Kpn*Ⅰ) | CTTGAATTCGAGCTCGGTACCACTTTGGGACTTT |
| *pAbAi-proRAP2.7-*F1*-*R (*Xho*Ⅰ) | ATACAGAGCACATGCCTCGAGGAGTTTTGATATAAT |
| *pAbAi-proRAP2.7-*F2*-*F (*Kpn*Ⅰ) | CTTGAATTCGAGCTCGGTACCAAATATTCTTACAAC |
| *pAbAi-proRAP2.7-*F2*-*R (*Xho*Ⅰ) | ATACAGAGCACATGCCTCGAGAAGTCTCCGGTAAAC |
| pGADT7-CiWRKY27-F (*Nde*Ⅰ) | GTACCAGATTACGCTCATATGATGGGTGAGAAATTT |
| pGADT7-CiWRKY27-R (*Xho*Ⅰ) | ACGATTCATCTGCAGCTCGAGTCAGCCGTGGTTGTC |
| pGEX-6P-1-CiRAP2.7-F (*Bam*HI) | TTCCAGGGGCCCCTGGGATCCATGTTGGATCTCAAT |
| pGEX-6P-1-CiRAP2.7-R (*Xho*Ⅰ) | GTCACGATGCGGCCGCTCGAGTGGTGGTGCCTGCGG |
| 62SK-CiWRKY27-F (*Bam*HI) | CGCTCTAGAACTAGTGGATCCATGGGTGAGAAATTT |
| 62SK-CiWRKY27-R (*Eco*RI) | GATAAGCTTGATATCGAATTCGCCGTGGTTGTCGCC |
| 62SK-CiRAP2.7-F (*Bam*HI) | CGCTCTAGAACTAGTGGATCCATGTTGGATCTCAAT |
| 62SK-CiRAP2.7-R (*Eco*RI) | GATAAGCTTGATATCGAATTCTGGTGGTGCCTGCGG |
| 0800-*proCiCAD7*-F (*Hind*III) | GTCGACGGTATCGATAAGCTTCATACCAAGTACTGG |
| 0800-*proCiCAD7*-R (*Bam*HI) | CGCTCTAGAACTAGTGGATCCGGAGTGGCTATTCAT |
| 0800-*proCiGSTF6*-F (*Hind*III) | GTCGACGGTATCGATAAGCTTGCTCGTTCGCAATAC |
| 0800-*proCiGSTF6*-R (*Bam*HI) | CGCTCTAGAACTAGTGGATCCTTGGCGCCCACCTTT |
| 0800-*proCiRAP2.7*-F1-F (*Hind*III) | GTCGACGGTATCGATAAGCTTACTTTGGGACTTT |
| 0800-*proCiRAP2.7*-F1-R (*Bam*HI) | CGCTCTAGAACTAGTGGATCCGAGTTTTGATATAAT |
| 0800-*proCiRAP2.7*-F2-F (*Hind*III) | GTCGACGGTATCGATAAGCTTAAATATTCTTACAAC |
| 0800-*proCiRAP2.7*-F2-R (*Bam*HI) | CGCTCTAGAACTAGTGGATCCAAGTCTCCGGTAAAC |
| YCE-CiWRKY27-F (*EcoR*Ⅰ) | ATGGGATCTACTAGTGAATTCATGGGTGAGAAATTT |
| YCE-CiWRKY27-R (*Kpn*Ⅰ) | TGGGTACATCCCGGGGGTACCGCCGTGGTTGTCGCC |
| YNE-CiWRKY27-F (*EcoR*Ⅰ) | ATGGGATCTACTAGTGAATTCATGGGTGAGAAATTT |
| YNE-CiWRKY27-R (*Kpn*Ⅰ) | TGGGTACATCCCGGGGGTACCGCCGTGGTTGTCGCC |
| YNE-CiRAP2.7-F (*EcoR*Ⅰ) | ATGGGATCTACTAGTGAATTCATGTTGGATCTCAAT |
| YNE-CiRAP2.7-R (*EcoR*Ⅰ) | TGGGTACATCCCGGGGGTACCTGGTGCCTGCGGCAG |
| cLUC-CiWRKY27-F (*Kpn*Ⅰ) | TACGCGTCCCGGGGCGGTACCATGGGTGAGAAATTT |
| cLUC-CiWRKY27-R (*Sal*I) | ACGAAAGCTCTGCAGGTCGACTCAGCCGTGGTTGTC |
| nLUC-CiWRKY27-F (*Kpn*Ⅰ) | ACGGGGGACGAGCTCGGTACCATGGGTGAGAAATTT |
| nLUC-CiWRKY27-R (*Sal*I) | CGCGTACGAGATCTGGTCGACTCAGCCGTGGTTGTC |
| nLUC-CiRAP2.7-F (*Kpn*Ⅰ) | ACGGGGGACGAGCTCGGTACCATGTTGGATCTCAAT |
| nLUC-CiRAP2.7-R (*Sal*I) | CGCGTACGAGATCTGGTCGACCTATGGTGGTGCCTG |

| **Table S2. List of probes used in the EMSA assay** | |
| --- | --- |
| GSTF6-probe | GATCGGTAGCTACTCTTCTTTGACTTCTCCACTATCCCTC |
| mutant-GSTF6-probe | GATCGGTAGCTACTCTTCTAAAAAATCTCCACTATCCCTC |
| CiCAD7-probe | TCTCGCCTTTTATCTTTGACTTTTTTTTTCTTTTTTAATG |
| mutant-CiCAD7-probe | TCTCGCCTTTTATCTAAAAAATTTTTTTTCTTTTTTAATG |
| RAP2.7-P3-probe | AAAATATTCTTACAACGTCAATAACGTAATAATTAAGCTA |
| mutant-RAP2.7-P3-probe | AAAATATTCTTACAAAAAAAATAACGTAATAATTAAGCTA |
| RAP2.7-P4-probe | GCTCAATTTTTGCTTGTCAAGTTTACCGGAGACTTCATC |
| mutant-RAP2.7-P4-probe | AAAAAATTTTTAAAAAAAAAAATTACCGGAGACTTCATC |

| **Table S3. The detail information of RNA-seq analysis** | | | | | | |
| --- | --- | --- | --- | --- | --- | --- |
| Sample | Total Raw Reads (M) | Total Clean Reads (M) | Total Clean Bases (Gb) | Clean Reads Q20 (%) | Clean  Reads Q30 (%) | Clean Reads Ratio (%) |
| A_27_1 | 43.82 | 43.04 | 6.46 | 97.47 | 93.2 | 98.22 |
| A_27_2 | 43.82 | 42.66 | 6.4 | 97.62 | 93.58 | 97.34 |
| A_27_3 | 43.82 | 42.77 | 6.42 | 97.75 | 93.91 | 97.61 |
| A_CK1 | 45.57 | 43.17 | 6.48 | 97.29 | 92.92 | 94.72 |
| A_CK2 | 45.57 | 43.16 | 6.47 | 97.47 | 93.39 | 94.7 |
| A_CK3 | 47.33 | 43.25 | 6.49 | 97.44 | 93.37 | 91.39 |
| B_27_1 | 43.82 | 42.86 | 6.43 | 97.62 | 93.56 | 97.82 |
| B_27_2 | 45.57 | 43.09 | 6.46 | 97.78 | 94 | 94.54 |
| B_27_3 | 43.82 | 42.76 | 6.41 | 97.65 | 93.67 | 97.59 |
| B_CK1 | 45.57 | 43.18 | 6.48 | 97.28 | 92.87 | 94.74 |
| B_CK2 | 43.82 | 42.98 | 6.45 | 97.58 | 93.46 | 98.09 |
| B_CK3 | 43.82 | 42.95 | 6.44 | 97.59 | 93.54 | 98.01 |

Note: “A” After cold stress, “B” Before cold stress, “CK” TRV:00 control plants, “27” TRV-*CiWRKY27* plants.

| **Table S4. The detail information of DAP-seq analysis** | | | | | | | | |
| --- | --- | --- | --- | --- | --- | --- | --- | --- |
| Sample | Raw Reads | Raw Bases | Clean Reads | Clean Bases | Clean Ratio | Q20 | Q30 | GC |
| IN_CiWRKY27 | 38112978 | 5716946700 | 37906714 | 5446324106 | 99.46% | 98.52% | 95.05% | 40.96% |
| IP_CiWRKY27 | 50407798 | 7561169700 | 50179102 | 7319903896 | 99.55% | 98.72% | 95.52% | 39.51% |

Note: “IN” Input, “IP” immunoprecipitation.

| **Table S5. The list of abbreviation** | |
| --- | --- |
| **Abbreviation** | **Full name** |
| AP2/ERF | AP2/Ethylene-Responsive Factor |
| C4H | Cinnamate 4-Hydroxylase |
| CAD | Cinnamyl Alcohol Dehydrogenase |
| CBF | C-repeat Binding Factor |
| CCR | Cinnamoyl-CoA Reductase |
| COMT | Caffeic acid *O*-methyltransferase |
| COR | Cold-Regulated Genes |
| C4H | Cinnamate 4-hydroxylase |
| DAP-Seq | DNA Affinity Purification Sequencing |
| DEGs | Different Expression Genes |
| GO | Gene Ontology |
| GST | Glutathione S-transferase |
| His | Histidine |
| KEGG | Kyoto Encyclopedia of Genes and Genomes |
| Leu | Leucine |
| MDA | Malondialdehyde |
| PAL | Phenylalanine Ammonia Lyase |
| ROS | Reactive Oxygen Species |
| SD | Synthetic Dropout |
| Trp | Tryptophan |
| Ura | Uracil |
| VIGS | Virus-Induced Gene Silencing |
